# A study on the car-following model for mountainous curves incorporating driving behavior characteristics

**DOI:** 10.1371/journal.pone.0352855

**Published:** 2026-07-15

**Authors:** Dong xiao Fu, Long jiao Zhang, Siyu Liu, Yuan Wang, Zhenya Ma, Yu Cao

**Affiliations:** 1 Yunnan Yunling Highway Science and Technology Co., Ltd. Kunming, Yunnan, China; 2 Faculty of Transportation Engineering, Kunming University of Science and Technology Kunming, Yunnan, China; Southwest Jiaotong University, CHINA

## Abstract

This study addresses the high accident rate on mountainous highways, driven by complex road alignments, harsh climatic conditions, and heterogeneous driving behaviors, aiming to enhance the accuracy of car-following behavior modelling. Using a typical mountainous curved section in Yunnan Province as the test case, drone-collected vehicle trajectory data were filtered using Kalman filtering to reduce noise. Subsequently, a K-Means algorithm optimized by differential evolution classified driving behaviors into three categories: aggressive, conservative, and standard. This revealed significant differences in speed, acceleration, and headway between distinct driving styles. To characterize curve dynamics, this study introduced a curve-radius parameter to enhance the Intelligent Driving Model (IDM). It calibrated it according to the rules for the three driving styles using genetic algorithms. Validation through macro-level error analysis and micro-level trajectory comparisons demonstrated that the improved model significantly enhances prediction accuracy for curve-following behavior while effectively adapting to diverse driving characteristics. This study pioneers the integration of driving behavior heterogeneity with curve geometry characteristics, providing a theoretical foundation for traffic flow simulation, safety assessment, and intelligent driving system design on mountain roads. It holds significant engineering value for reducing the risk of following-distance accidents and optimizing traffic management in mountainous regions.

## 1 Introduction

Rapid socioeconomic development has resulted in a significant increase in the number of motor vehicles and drivers, intensifying conflicts among people, vehicles, and roads. The mountainous road environment is characterized by its complexity and intricacy. Road conditions are heavily influenced by topography and terrain, leading to several distinct features: Alignment designs often include complex structures, such as continuous steep gradients and compound curves. These features frequently create blind spots, contrib uting to high-risk areas. Pavement conditions are strongly influenced by the natural environment, leading to unstable skid resistance. During adverse weather conditions, such as rain or snow, road surfaces can become extremely slippery, further increasing the risks associated with driving. Additionally, the climate in mountainous areas is notoriously unpredictable. Visibility can fluctuate drastically due to weather conditions, further complicating matters. The threat of geological hazards, such as mudslides, further complicates safety challenges encountered during road travel. Furthermore, traffic conditions in mountainous regions are relatively chaotic. It is characterized by mixed traffic patterns, a high proportion of heavy trucks, and a dense concentration of special structures, such as bridges and tunnels. These factors collectively complicate driving conditions.

In mountainous regions characterized by complex topography, advancing highway construction encounters multiple challenges. On one hand, constraints arise from geological and topographical features, limitations in vehicle technology, and insufficient risk awareness among drivers. On the other hand, alignment design standards restrict mountain roads, which commonly feature concentrated safety hazards such as continuous steep gradients, compound curves, and blind spots. Additionally, harsh mountain weather exacerbates these issues, as road surfaces can become extremely slippery during rain or snow. Consequently, these factors collectively lead to a higher frequency of accidents on mountain roads than in plains regions [1]. The risks associated with driving in these special sections increase significantly, severely disrupting daily life and negatively impacting social stability. Furthermore, multi-vehicle collisions on mountain highways tend to result in more severe consequences compared to single-vehicle accidents [2]. As the core determinant of vehicle safety, precise driving decisions hold particular research value in the unique traffic environment of mountainous curves. Car-following behavior, a typical driving pattern in this context, serves as a key indicator of drivers’ dynamic response characteristics and is a central research subject in micro-traffic flow theory. From a systems theory perspective, the tailgating process on mountain curves fundamentally represents a dynamic interaction problem within the typical human-vehicle-road-environment system. In this context, drivers’ behavioral characteristics and decision-making mechanisms exert a dominant influence. The rationality of highway alignment design not only affects economic investment in road construction but also directly influences driving smoothness, road traffic efficiency, driver operational behavior, and fatigue levels during driving [3].

Regarding road alignment, Bashar et al. utilized natural driving research data to investigate the relationship between speed reduction and horizontal curve collision frequency. They proposed a new safety performance model. The findings indicate that speed reduction is a significant factor influencing curve safety performance [4]. When drivers navigate curves on mountainous roads, variations in speed significantly affect their performance. Sil et al. developed artificial neural network (ANN) and genetic algorithm (GA) models to predict free-flow and 85th-percentile speeds on major urban arterial roads in Jordan. Their research revealed that parameters such as horizontal curve radius, angle, and arc length significantly influence driving speeds [5]. To address these issues, some scholars have proposed corresponding measures for improvement. Babić D et al. designed scenarios with different combinations of lane markings. They collected driving data on curves using a driving simulator to verify the impact of various marking combinations on driver behavior, particularly before and after hazardous curves. Their findings indicate that low-cost road marking measures can effectively reduce driving speeds and enhance driving stability [6].

Driving behavior is inherently a multidimensional information-processing system that involves the dynamic interaction among environmental perception, risk decision-making, and control execution. Most studies focus on the relationship between drivers and geometric indicators. DARZI et al. integrated driver characteristics, vehicle kinematics, and physiological measurements. Their findings demonstrate that external factors, such as road geometry, influence drivers’ physiological traits [7]. Feng et al. conducted real-vehicle experiments to analyze the effects of urban longitudinal gradients on drivers’ heart rates and speeds. They found that variations in heart rate effectively reflect psychological changes in drivers [8]. Additionally, some scholars have examined this topic from the perspective of driver behavior. In their research on road geometry, George et al. employed computer animation techniques. Their results reveal significant differences in the effectiveness of horizontal and vertical road alignments when providing visual guidance to drivers [9].

The car-following model serves as a fundamental theoretical tool in traffic flow micro-simulation, revealing the operational patterns of transportation systems by establishing longitudinal interaction mechanisms between vehicles. In the context of research on mechanical characteristics, Zhu et al. proposed a car-following model that examines the effects of friction coefficients, curvature radii, and gradients on traffic flow stability, accounting for the characteristics of curved road surfaces. The findings indicate that traffic flow stability varies significantly across different road conditions [10]. In studies focused on curve geometry, Zhang et al. investigated the impact of small-radius curves on driving behavior. They introduced the psychological factor of visual characteristics and proposed a model based on the driver’s expected visual angle. Through linear stability analysis and numerical simulation, they validated the model’s effectiveness, leading to significant improvements in traffic flow stability and safety on small-radius curves [11]. Current research on factors influencing driver following behavior highlights substantial heterogeneity within the driving population. This heterogeneity arises partly from intrinsic differences among drivers in terms of skills, reaction times, and other personal attributes. Furthermore, when drivers encounter various traffic scenarios—such as congested urban roads, smooth highways, or winding mountain roads—their driving behaviors vary markedly, reflecting heterogeneity shaped by external contexts. Upon receiving road information, drivers respond immediately and manipulate their vehicle controls accordingly. Michaels established a driving decision model based on dynamic constraints of the field of view [12]. Building on this foundation, Grey et al. developed the following algorithm that incorporates visual occlusion effects, significantly enhancing behavioral prediction accuracy in curved road scenarios [13]. To address the mechanisms driving errors, Stanton et al. developed a latent classification model applicable to intelligent driving [14]. Young revealed a dose-response relationship between distraction and operational errors [15].

Current research on curve driving, driving behavior, and following models has made progress in various areas. However, significant gaps remain in multidimensional integration and the exploration of interaction mechanisms. Notably, the relationship between curve-following behavior and driver heterogeneity warrants further investigation. The heterogeneity of driving behavior has become a key focus in micro-level traffic flow studies. Numerous studies have confirmed that driving styles are classifiable and exert a significant impact on car-following models, based on diverse datasets and methodologies. For instance, Yang Ze [16] categorized driving styles into aggressive, conservative, and moderate types based on NGSIM trajectory data. He demonstrated that distinct model parameters can represent these different styles. Qiu Xiaoyun [17] utilized K-means clustering to classify driving styles into four categories, highlighting variations in perceptual-reaction characteristics. Moreover, studies indicate that human factors—including driver memory effects [18,19], perceptual-reaction characteristics [20], and asymmetric responses to speed differentials [21]—are crucial sources of heterogeneity in driving behavior. These factors can exhibit different mechanisms of influence u under specific conditions, such as adverse weather, as shown in the research by Yang LH et al. [22]. Together, these studies establish the theoretical foundation for constructing the following models that consider driving behavior characteristics. Future research on the mechanisms underlying the interaction between curve-following behavior and driver heterogeneity must transcend traditional single-perspective limitations. It should aim to develop a comprehensive analytical model that encompasses road environments, vehicle dynamics, and driver behavior. This paper investigates vehicle-following behavior on mountainous highway curves, using empirical data to examine operational patterns under these conditions. Ultimately, the goal is to construct a following model that integrates driver behavioral heterogeneity with complex environmental factors.

**Fig 1:**
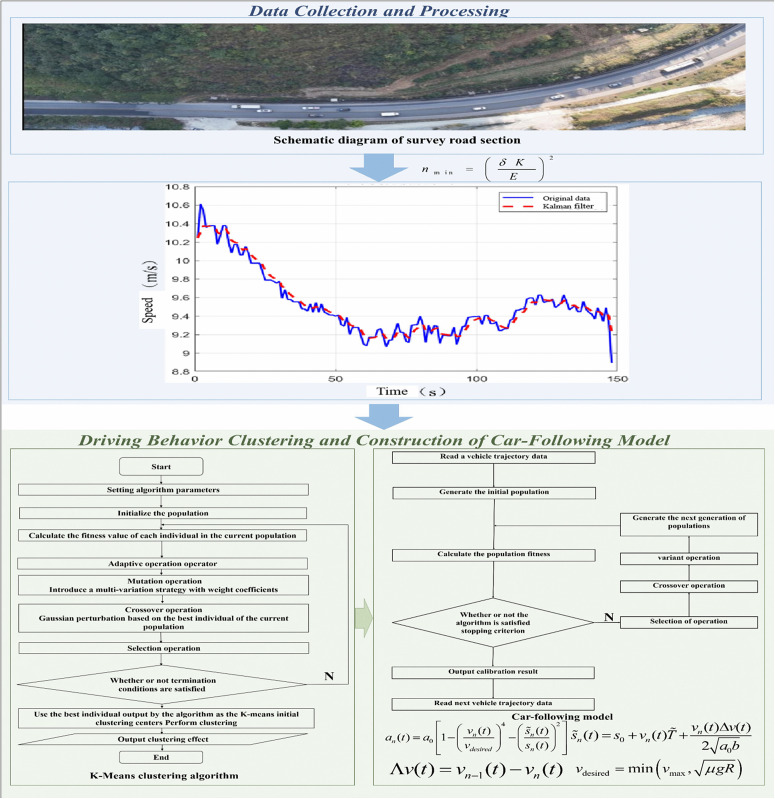
Research Framework.

To bridge gaps in existing research, this study focuses on typical curved road segments in mountainous areas of Yun County, Lincang City, Yunnan Province. Vehicle trajectory data were collected using Unmanned Aerial Vehicles (UAVs) and denoised with the Kalman filter. By extracting 12 key driving-behavior feature parameters, driving behaviors were accurately classified using the K-Means clustering algorithm optimized with Differential Evolution (DE). Furthermore, using the Intelligent Driver Model (IDM) as the basic framework, a mountainous-curve car-following model was developed by introducing curve-radius parameters to refine the desired speed and by integrating the heterogeneity of driving behaviors. This model establishes a coupling mechanism between the curve radius correction term and driving styles to more realistically reflect the speed-adaptation behavior of different drivers on curves. Finally, based on the scenario innovation of complex mountain alignments, the model parameters for different driving styles were calibrated using Genetic Algorithms (GA). The model’s prediction accuracy and adaptability were systematically validated across two dimensions: macroscopic Root Mean Square Error (RMSE) and microscopic vehicle trajectory comparison. This study has practical significance for establishing the correlation between microscopic behaviors and macroscopic traffic characteristics and for improving traffic safety management in curved sections of mountainous roads. The research framework is shown in Fig 1.

## 2 Data collection and processing

### 2.1 Test section

Mountainous highway alignments exhibit typical composite geometric characteristics. Their spatial layout integrates three key elements: straight sections, transition curves, and circular curve units. During travel on consecutive sections, vehicle forces are relatively straightforward, the direction of travel is clear, and driving operations are uncomplicated. As a result, vehicles generally achieve higher speeds with minimal fluctuations in measured speed. Curve design typically comprises two fundamental forms: transition curves and circular curves. These designs aim to ensure that vehicles can smoothly alter their direction while maintaining safety. When traversing a single curved section, vehicles first enter a transition curve to facilitate a smooth change in alignment. They then proceed into the circular curve segment and finally exit the curve smoothly via another transition curve.

Considering the characteristics of mountainous road curves and vehicle operating conditions, this paper integrates Baidu Maps data with field survey data. Data were collected from a typical curved mountainous section in Yun County, Lincang City, Yunnan Province. This section is classified as a mountainous highway, featuring pronounced curves and a moderate traffic volume. The study section, Yun County Highway (Yun County-Lincang Highway), spans K2677 to K2677 + 350 and is located in the northwestern plateau region of Yunnan Province. It offers good visibility and consists of two lanes. The speed limit on this highway is set at 16.67 m/s. Roadside guardrails are installed for collision protection; however, there are no central separation facilities. The curve design is notably unique, making driving more difficult due to the road’s varying curvature. As a result, overtaking is generally unfeasible, and vehicles are more susceptible to car-following behavior.

### 2.2 Data collection

**Fig 2:**
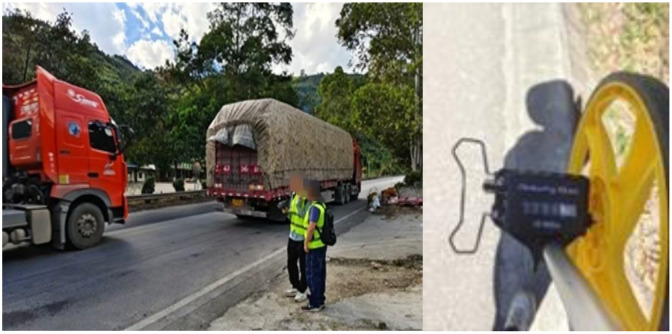
Field research map.

**Fig 3:**
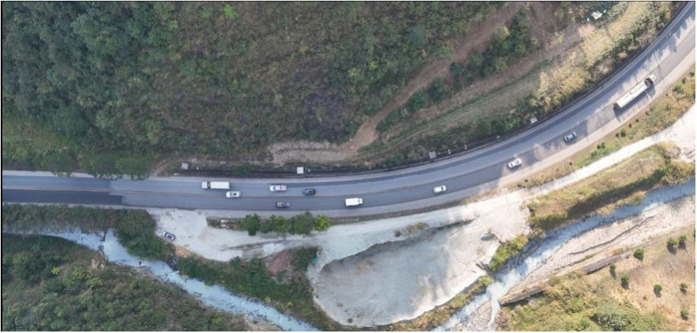
Schematic diagram of survey road section.

According to the survey requirements, a field survey and inspection were first conducted at the preliminarily selected aerial survey locations. This involved preliminary assessments of road alignment, vehicle speed, and the surrounding environment at each point to identify suitable road segments for aerial data collection. After determining the collection locations, the survey team carried out on-site data collection from October to December 2023. A DJI Air 3 drone was used for aerial observation, with the shooting parameters set to 4K (3840 × 2160) at 60 fps. Fig 2 shows a field survey photograph. To obtain comprehensive and accurate vehicle driving behavior data, the flight altitude and aerial camera angle were adjusted multiple times to ensure stable overhead shooting, with example results shown in Fig 3.

**Fig 4:**
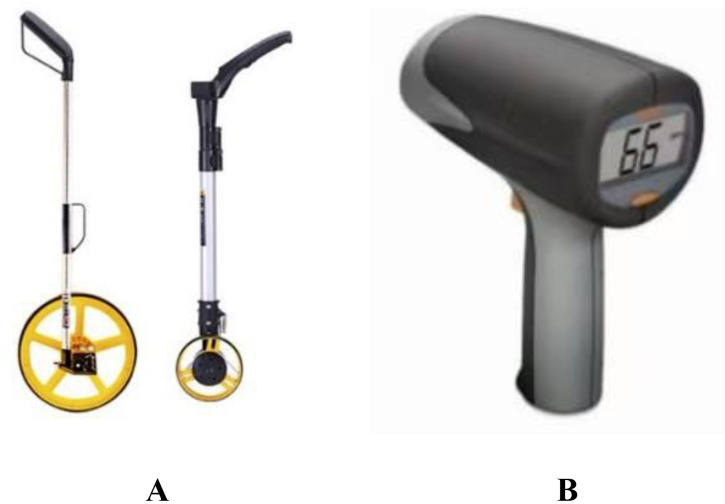
Auxiliary measuring instruments. A: Mechanical distance measuring wheel; **B:** Mobile radar speed gun.

In addition, as shown in Fig 4, to verify data accuracy, a mechanical measuring wheel (Deli, 0304) and a mobile radar speed gun (Bushnell, 01911) were used as auxiliary data-collection devices. The mechanical measuring wheel can measure the distance between any two observation points captured by the drone, with a measurement accuracy of ±0.5%; the radar speed gun can detect vehicle speed with a measurement accuracy of ±1.0 MPH to ±2.0 KPH.

No specific administrative permits or ethics approval were required for this study. The research was conducted on a public highway in Yun County, Lincang City, Yunnan Province, China. This road section is an open-access roadway with no special entry restrictions. All data were collected in compliance with relevant regulations through non-invasive aerial observation of vehicles in public space. No personal privacy information (e.g., driver faces, license plate numbers, etc.) was recorded or retained.

### 2.3 Data processing

To achieve precise capture of vehicle trajectories, a multi-scale KCF algorithm was employed to extract full-sample trajectories from video data. This approach effectively identified vehicle movement paths within curved sections. To ensure that observation accuracy aligns with preset requirements and that the sample size is sufficient to reflect actual conditions on mountainous highway curves, the minimum required sample size for the observation section was determined based on statistical principles as follows:


$$\begin{array}{c} n_{min}={\left(\frac{\delta K}{E}\right)}^{\text{2}} \end{array}$$ (1)

In the formula, $n_{min}$ represents the minimum sample size for vehicle speed; *δ* denotes the standard deviation of vehicle speed, which is set at 3.056 m/s in this study; *E* indicates the permissible error for vehicle speed observation, typically set at 0.556 m/s; and *K* represents the confidence level. At a 90% confidence level, *K* equals 14—calculations using Equation (1) yield a minimum sample size of 53 vehicles. Among the total data samples, 496 vehicle trajectories were recorded, with valid trajectories comprising 95.16% of the total, thereby meeting the sample size requirement.

**Fig 5:**
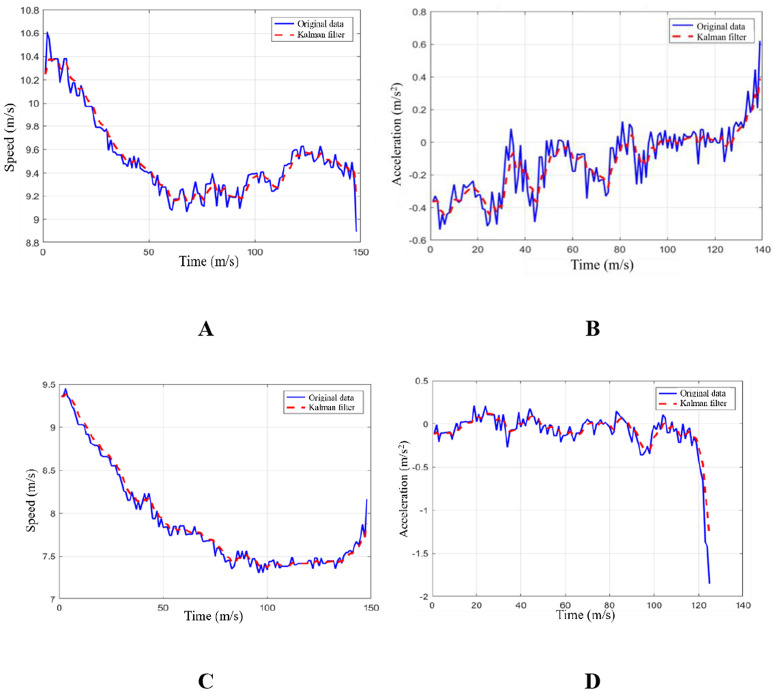
Selected sample speed, acceleration smoothing effect diagram. **A:** Denoising effect of conventional velocity data; **B:** Denoising effect of conventional acceleration data; **C:** Denoising effect of controlled velocity data; **D:** Denoising effect of controlled acceleration data.

To address potential noise interference in the raw trajectory data, Kalman filtering was used to reduce noise. Results are illustrated in Fig 5, where blue represents the raw data, and red indicates the filtered data. A comparison shows that the filtered velocity and acceleration curves are smoother. This filtering effectively eliminates noise and enhances the stability, integrity, and accuracy of the data.

Furthermore, the curve radius is a critical metric for assessing the degree of horizontal curvature. Its magnitude directly dictates vehicle speed, stability, and operational safety during cornering maneuvers. In scenarios where the center of the circle is unknown, determining the radius of the curve poses significant technical challenges. To address this, the radius of the circular curve can be calculated by measuring the arc length and its corresponding chord length, using established formulas [23]. The specific governing equations are as follows:


$$\begin{array}{c} S=r\theta \end{array}$$ (2)

$$\begin{array}{c} L=2rsin(\theta /2) \end{array}$$ (3)

By solving Equations (2) and (3), the following expression is obtained:


$$\begin{array}{c} S=2r\times arcsin(L/2R) \end{array}$$ (4)

**Fig 6:**
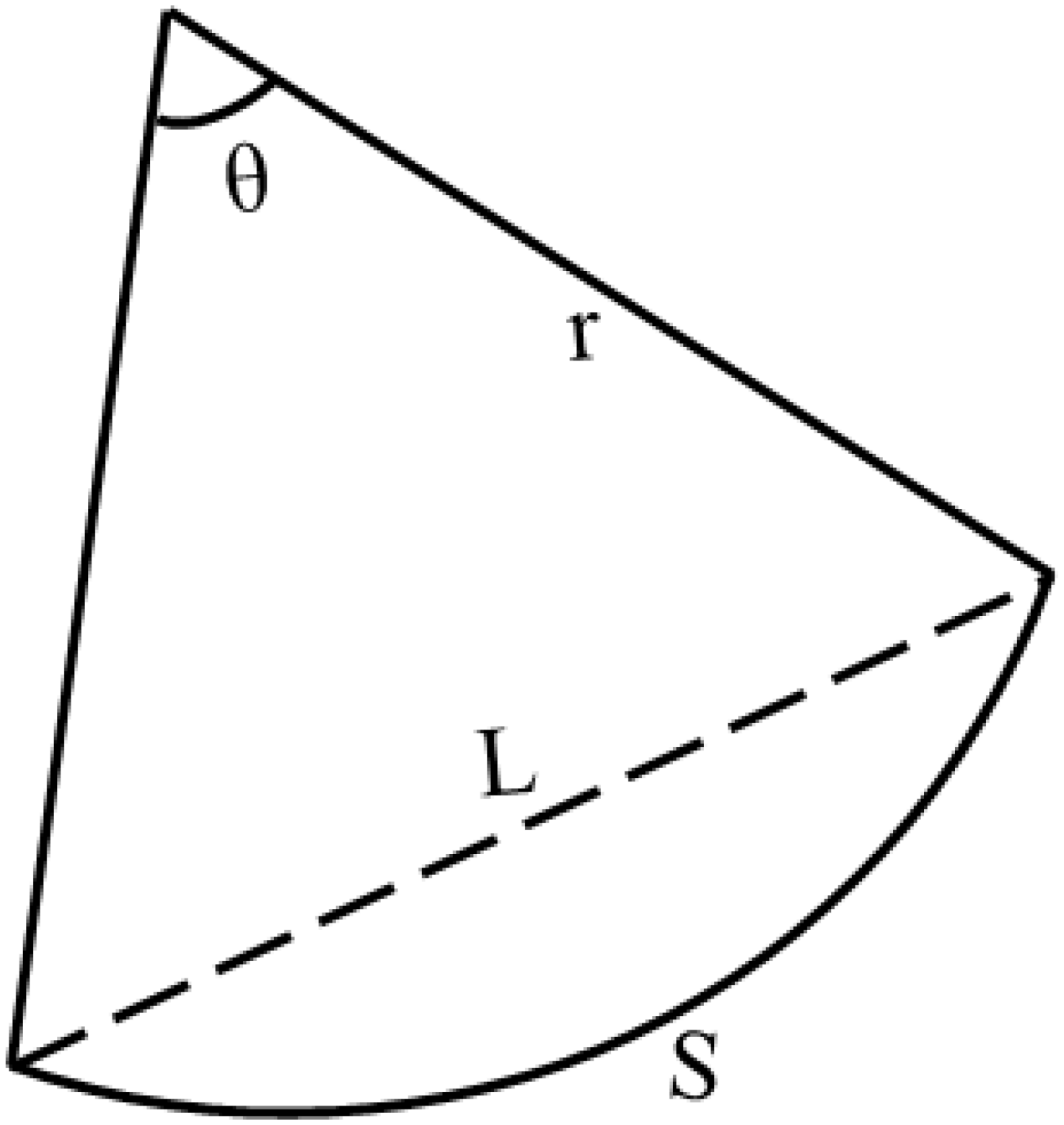
Diagram of arc length and chord length of bend.

In the governing equations, *S* and *L* represent known quantities, where *S* denotes the arc length of the curve and *L* denotes the corresponding chord length. The variables *r* and *θ* are unknowns, where *r* is the radius of the circular curve, and *θ* is the central angle subtended by the arc. To minimize calculation errors in determining the curve radius, a sampling method was employed. Specifically, *x* groups of data (*x* ∈ [0, 100]) were extracted. For each group, the arc length and its corresponding chord length were measured using CAD software. Subsequently, the radius of the circular curve was calculated in MATLAB using Equation (4). The geometric relationship between the arc length and the chord length is illustrated in Fig 6.

After completing the calculation of the circular curve radius of the bend, the maximum and minimum values were discarded from the obtained data, and the average of the remaining intermediate values was calculated. The circular curve radius of the bend was finally determined to be 154 m.

### 2.4 Analysis on operational characteristics of drivers’ car-following behavior

**Fig 7:**
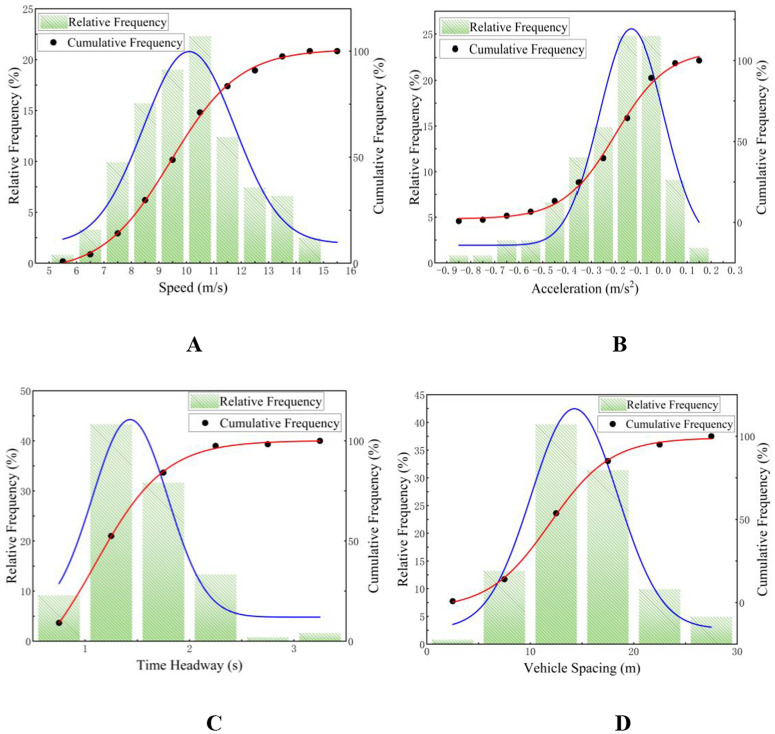
Statistical Frequency Distribution Diagram of Vehicle Speed, Acceleration, Time Headway, and Vehicle Spacing on Mountainous Curves. **A:** Velocity statistical frequency distribution diagram; **B:** Acceleration statistical frequency distribution diagram; **C:** Statistical frequency distribution of time headway; **D:** Statistical frequency distribution of headway.

To comprehensively grasp the basic laws of vehicle operation on mountainous curved sections, valid car-following events are extracted from car-following data in this paper. Statistical methods are used to analyze the indicators of speed, acceleration, vehicle spacing, and time headway for the full sample data. Relative frequency distribution histograms, cumulative frequency curves and fitting results are plotted, as illustrated in Fig 7. On this basis, the car-following behavioral characteristics of vehicles on curved sections of mountainous highways are analyzed, the car-following features are summarized, the variation laws of microscopic driving behavioral characteristics of vehicles in curves are explored, and the traffic operation characteristics of mountainous highway curved sections are clarified.

According to the statistical frequency distribution in Fig 7, the operational characteristics of vehicles on mountainous curves exhibit a clear unimodal concentration. The peak of the speed distribution occurs near 10–11 m/s. The peak acceleration is approximately −0.2 m/s^2^, indicating that most drivers pass through the curve slightly decelerating and select a relatively safe and comfortable speed range. Cases of excessively high or low speed as well as sharp acceleration and deceleration rarely occur. The frequency of vehicle spacing reaches. The peak time headway appears around 1 s, while the frequency of excessively small or large spacing and headway isa maximum at approximately 15 m, with a peak time headway of around 1 s. In contrast, the frequencies of excessively small or large spacing and headway are relatively low. This reflects that drivers tend to maintain moderate car-following distance and time headway on mountainous curves to balance driving safety and road traffic efficiency. The results reveal that drivers adjust vehicle spacing and speed more cautiously to ensure driving safety during car-following on mountainous curves, and that their car-following behavior exhibits greater restrictiveness, delay, and transferability. Therefore, it is necessary to research car-following models that account for driving behavioral characteristics.

**Fig 8:**
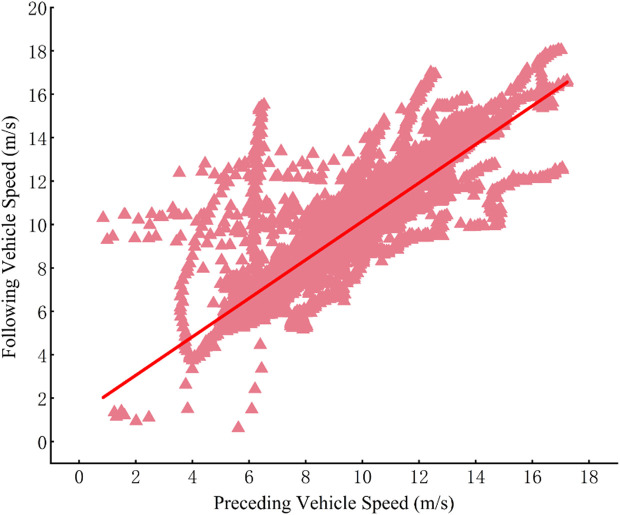
Front and rear vehicle speed pairs.

As shown in Fig 8, taking the speed of the leading vehicle as the horizontal axis and the speed of the following vehicle as the vertical axis, speed pairs (*V*_*leading*_, *V*_*following*_) are constructed. It can be observed that the point set composed of speed pairs is symmetric about the ray *V*_*leading*_ *= V*_*following*_, and most points are concentrated on this ray.

**Fig 9:**
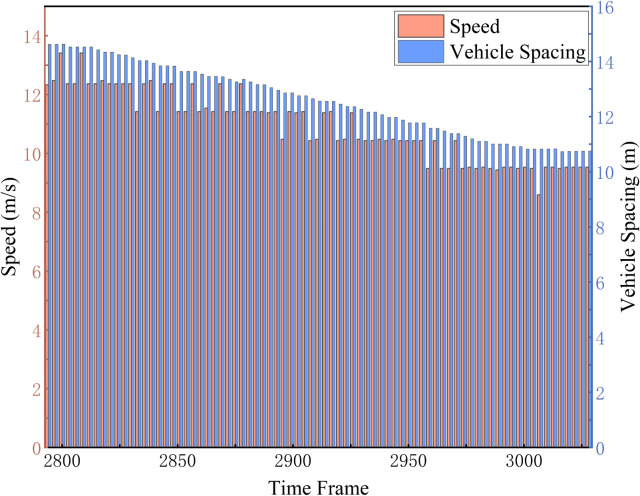
Speed-headway change example diagram.

In addition, the relationship between car-following speed and vehicle spacing is plotted in Fig 9. It can be found that both speed and vehicle spacing show an overall downward trend with the increase of time frames. This indicates a consistent variation trend between speed and vehicle spacing; that is, the speed decreases synchronously with the reduction of vehicle spacing.

In summary, vehicle car-following behavior is prevalent in medium-density traffic flow. Under the free-flow state, characterized by low density and large intervals between vehicles, and the interaction between leading and following vehicles is extremely weak. The interaction between leading and following vehicles is extremely weak, with no car-following phenomena. Once traffic congestion occurs, traffic density rises sharply and vehicle spacing restricts drivers’ operational choices. In contrast, the influences of drivers’ personal traits and vehicle performance on car-following maneuvers are relatively insignificant. During the car-following process, drivers to consider factors such as their own speed, the speed of the leading vehicle, and the distance between the two vehicles to make driving decisions, including following, lane changing, and overtaking.

## 3 Method

### 3.1 DE-K-means-based method for classifying driving styles in curves

#### 3.1.1 Feature vector extraction related to driving style.

While driving on low-grade mountain roads, a driver’s state is influenced by the interaction of multiple factors. Externally, environmental conditions, variations in road alignment, traffic management rules, and road conditions continuously relay dynamic information to the driver. This information initially affects the driver’s psychological state, making them susceptible to emotional fluctuations, such as tension, when faced with uncertainties like changes in weather or road alignment. These psychological changes further lead to physiological alterations, including increased heart rate, dilated pupils, and altered visual perception. The driver’s combined physical and mental state directly impacts their operational actions, adjusting vehicle conditions and driving dynamics through pedal inputs and steering control. Ultimately, these driving behaviors also provide feedback to the driver. In terms of driving behavior characteristics, Lee et al. [24], Lu et al. [25], and Magana et al. [26] have characterized these behaviors using metrics such as speed, lateral acceleration, pedal force, and steering wheel angle.

Driving behavior characteristics represent the multidimensional dynamic responses within the driver’s perception-cognition-decision-execution chain. This encompasses all operational actions the driver performs during vehicle operation. Driving behavior is influenced by a complex interplay of factors, including the driver’s personality traits, the vehicle’s performance, the driver’s current mental state, and the surrounding environment. Throughout the driving process, the driver must continuously and integrate perceptual information from multiple modalities in real time. This requires not only close attention to the dynamic traffic environment but also constant monitoring of key parameters such as vehicle speed and trajectory. By effectively processing the constantly changing external environmental information, the driver can promptly adjust the vehicle’s operational state, thereby ensuring driving safety. A comprehensive review and analysis of relevant literature [27] was conducted, taking into account the characteristics of vehicle-following behavior. An in-depth study was performed on the extracted following segment trajectory data. Following careful screening, 12 feature parameters that accurately reflect driving behavior characteristics were ultimately identified, as shown in Table 1.

**Table 1:** Statistical table of parameters.

Serial No.	Parameter Name	Serial No.	Parameter Name
X1	Average Lateral Speed	X7	Average Acceleration
X2	Maximum Lateral Speed	X8	Maximum Acceleration
X3	Average Longitudinal Speed	X9	Average Headway
X4	Maximum Longitudinal Speed	X10	Maximum Headway
X5	Average Speed	X11	Average Headway Duration
X6	Maximum Speed	X12	Maximum Headway Duration

This study selected 12 driving behavior characteristic parameters to capture differences in driving behavior fully. Although these parameters reflect driver control characteristics from multiple dimensions, considerable information overlap and weight discrepancies exist among them. Direct modelling with the original feature set would not only increase computational complexity but also introduce risks—such as slower convergence, reduced generalization, and lower prediction accuracy—due to multicollinearity. Therefore, this study aims to apply feature optimization and dimensionality reduction to remove redundant features and extract core discriminative factors while preserving the completeness of the original data.

#### 3.1.2 Dimensionality reduction of driving behavior characteristics based on factor analysis.

Before constructing the classification model for driving behavior characteristics, this study employed nonparametric statistical methods to assess nonlinear correlations among the features. The Spearman rank correlation coefficient was used to assess multicollinearity in the feature set. Its calculation is based on the covariance relationship of the original data after rank ordering, effectively identifying redundant information in the feature matrix and providing a quantitative basis for constructing a reduced feature subset in subsequent steps. The correlation coefficient is computed as follows:

Suppose there are n pairs of observed variables *x*_*i*_ and *y*_*i*_*,* which are sorted from largest to smallest. After sorting, the positions of the original variables are denoted as *x*_*i*_*’* and *y*_*i*_*’*, respectively, which serve as their ranks. The difference between the ranks of *x*_*i*_ and *y*_*i*_ is then:


$$\begin{array}{c} d_{i}=\overset{\prime }{\underset{i}{x}}-\overset{\prime }{\underset{i}{y}} \end{array}$$ (5)

If there are no tied ranks, then


$$\begin{array}{c} \rho_{s}=1-\frac{6\sum \overset{\text{2}}{\underset{i}{d}}}{n(n^{\text{2}}-1)} \end{array}$$ (6)

If there are tied ranks, it is necessary to calculate the Pearson linear correlation coefficient among the ranks:


$$\begin{array}{c} \rho_{s}=\frac{\sum_{i}(x_{i}-\overset{―}{x})(y_{i}-\overset{―}{y})}{\sqrt{\sum_{i}(x_{i}-\overset{―}{x}{)}^{\text{2}}(y_{i}-\overset{―}{y}{)}^{\text{2}}}} \end{array}$$ (7)

where $\overset{―}{x}$ and $\overset{―}{y}$ are the means of the observed variable sequences.

**Fig 10:**
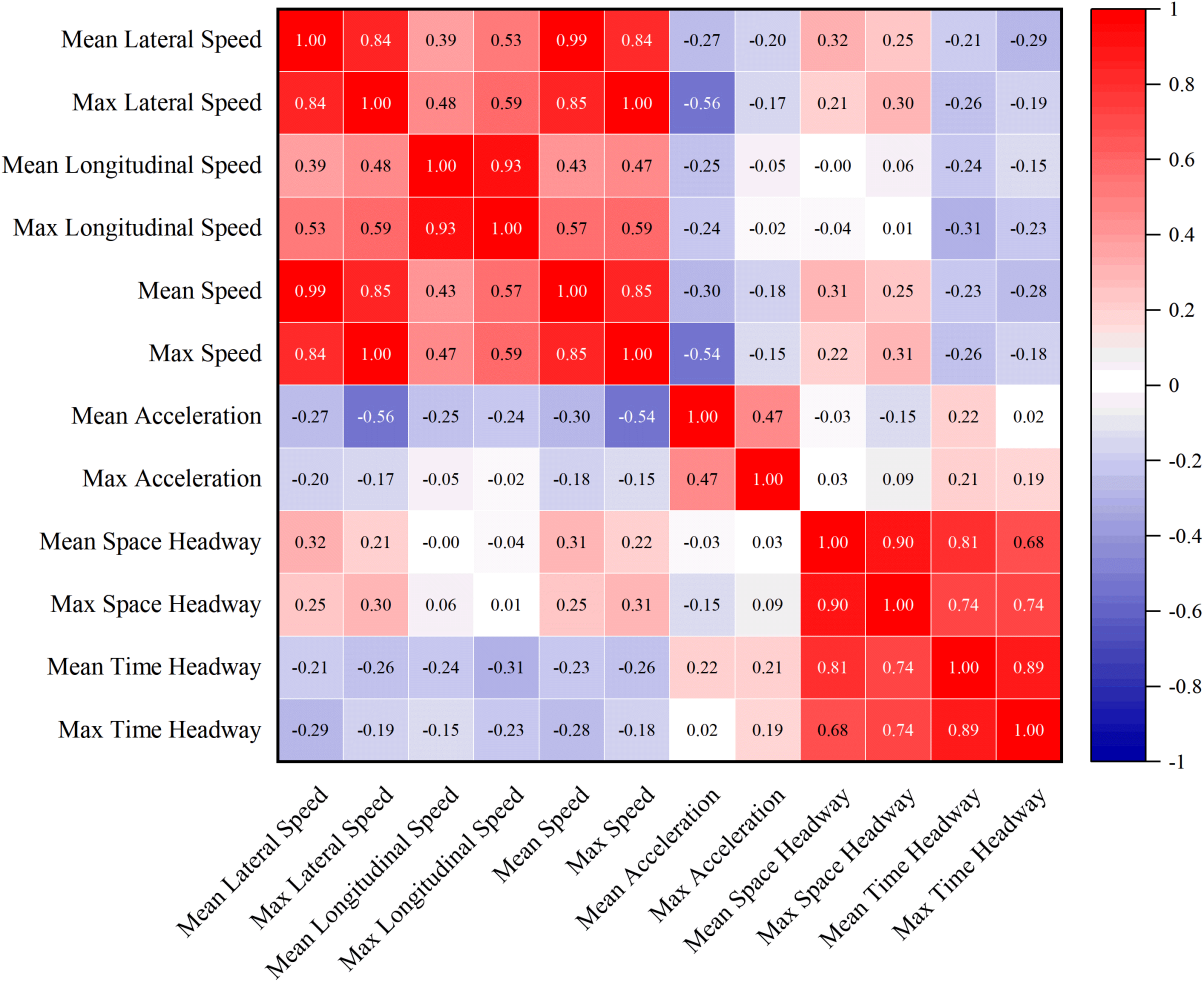
Correlation coefficient heat map.

Based on the Spearman rank correlation coefficient, a heatmap of eigenvector Spearman rank correlation coefficients for drivers’ driving behavior characteristics is plotted, as shown in Fig 10. It can be seen that the absolute values of the rank correlation coefficients among all eigenvectors are greater than 0.6, indicating a correlation exists between most vectors. Therefore, dimensionality reduction processing is required to meet the evaluation requirements.

Factor analysis was performed in SPSS based on the 12 screened driving behavior characteristic parameters. Factor analysis is applicable only when there are high correlations among variables. As indicated by the previous Pearson correlation analysis, significant correlations existed among most variables. The screened data were processed and subsequently subjected to factor analysis in SPSS. As shown by the KMO and Bartlett’s test results in Table 2, the KMO value was 0.685, and the significance (Sig) value of Bartlett’s sphericity test was 0.000, which was lower than the significance level of 0.05. The results verified significant correlations among the characteristic parameters, further demonstrating that the selected parameters were suitable for factor analysis.

**Table 2:** Correlation test.

KMO value		0.685
Bartlett’s Test of Sphericity	Approximate Chi-square	2650.689
	df	66
	p-value	0

Table 3 shows the rotated component matrix, which presents the complex correlations between a series of variables and factors. By carefully examining the matrix, the numerical values of each variable for different factor dimensions can be clearly identified. Through in-depth analysis of the data in each column, the maximum value is selected, in accordance with rigorous statistical principles. Within the framework of factor analysis, extracting the maximum value identify which factor has the strongest correlation with each variable. This is because the factor corresponding to the maximum value has the strongest explanatory power for the variable, indicating that the information contained in the variable is reflected to the greatest extent on this factor. It enables an in-depth understanding of the internal relationships among variables and factors, thereby facilitating data analysis and subsequent research decision-making.

**Table 3:** Factor load coefficient table after rotation.

Name	Factor Loading Coefficient			
	Factor 1	Factor 2	Factor 3	Factor 4
Mean Lateral Speed	0.957	0.020	0.148	−0.062
Maximum Lateral Speed	0.905	0.039	0.269	−0.210
Mean Longitudinal Speed	0.262	−0.032	0.916	−0.056
Maximum Longitudinal Speed	0.434	−0.103	0.853	0.009
Mean Speed	0.951	0.008	0.189	−0.067
Maximum Speed	0.908	0.036	0.266	−0.196
Mean Acceleration	−0.187	−0.091	−0.314	0.820
Maximum Acceleration	−0.196	0.193	0.277	0.744
Mean Headway Distance	0.292	0.909	−0.102	0.108
Maximum Headway Distance	0.206	0.912	0.041	0.023
Mean Time Headway	−0.308	0.900	−0.173	0.072
Maximum Time Headway	−0.406	0.653	0.266	−0.403

Based on the factor analysis results, the characteristic loadings and naming basis of the four common factors are as follows: the first common factor exhibits high loadings on mean lateral speed (0.957), maximum lateral speed (0.905), mean speed (0.951), and maximum speed (0.908), which mainly reflect the lateral motion characteristics of the vehicle and are thus named the lateral motion factor; the second common factor has significant loadings on mean headway distance (0.909), maximum headway distance (0.912), mean time headway (0.900), and maximum time headway (0.653), comprehensively characterizing the safety distance between vehicles and defined as the safety spacing factor; the third common factor shows prominent loadings on mean longitudinal speed (0.916) and maximum longitudinal speed (0.853), reflecting the motion characteristics of vehicles in the driving direction and named the longitudinal motion factor; the fourth common factor presents the highest loadings on mean acceleration (0.820) and maximum acceleration (0.744), which are closely associated with drivers’ braking and speed adjustment behaviors under car-following conditions, and is therefore defined as the acceleration behavior factor.

Let F1、F2、F3and F4 denote the lateral motion factor, safety spacing factor, longitudinal motion factor, and acceleration behavior factor, respectively. Based on Table 4, the expressions for the four common factors are obtained as follows:

**Table 4:** Component score coefficient.

Name	Component			
	Component 1	Component 2	Component 3	Component 4
Mean Lateral Speed	0.266	0.012	−0.094	0.047
Maximum Lateral Speed	0.215	0.017	−0.019	−0.055
Mean Longitudinal Speed	−0.097	−0.004	0.517	0.045
Maximum Longitudinal Speed	−0.026	−0.027	0.449	0.101
Mean Speed	0.256	0.008	−0.068	0.046
Maximum Speed	0.217	0.016	−0.020	−0.045
Mean Acceleration	0.070	−0.026	−0.104	0.551
Maximum Acceleration	−0.050	0.073	0.262	0.539
Mean Headway Distance	0.125	0.310	−0.106	0.104
Maximum Headway Distance	0.063	0.310	−0.006	0.045
Mean Time Headway	−0.053	0.303	−0.039	0.022
Maximum Time Headway	−0.209	0.217	0.219	−0.300


$$\begin{array}{c} \left\{ \begin{array}{c} @l@F_{\text{1}}=0.266x_{\text{1}}+0.251x_{\text{2}}+\cdots -0.053x_{11}-0.209x_{12}\\ F_{\text{2}}=0.012x_{\text{1}}+0.017x_{\text{2}}+\cdots +0.303x_{11}+0.217x_{12}\\ F_{\text{3}}=-0.094x_{\text{1}}-0.019x_{\text{2}}+\cdots -0.039x_{11}+0.219x_{12}\\ F_{\text{4}}=0.047x_{\text{1}}-0.055x_{\text{2}}+\cdots +0.022x_{11}-0.300x_{12} \end{array}\right. \end{array}$$ (8)

**Fig 11:**
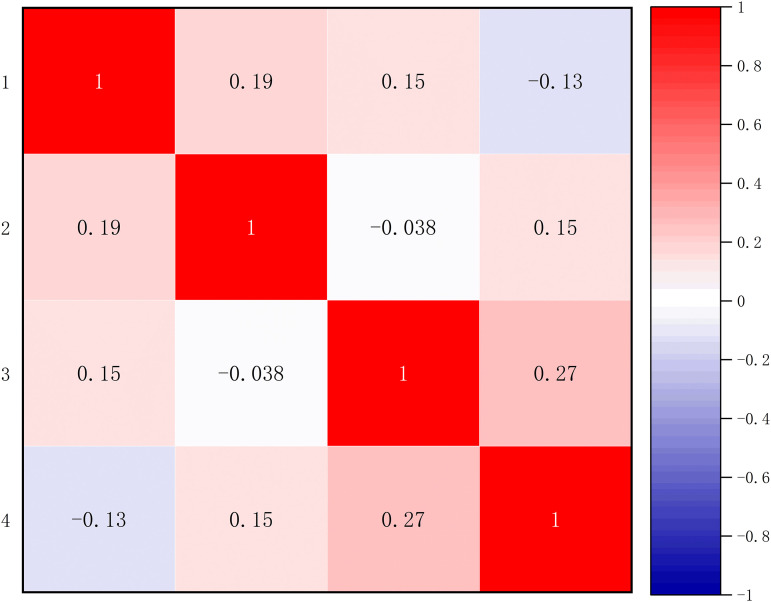
Spearman rank correlation coefficient heat map (after factor analysis).

After dimensionality reduction of the driving behavior characteristic parameters via factor analysis, correlation analysis was conducted on the resulting factors, as shown in Fig 11. Calculations based on the Spearman rank correlation coefficient for the vectors obtained after factor analysis reveal that the absolute values of the rank correlation coefficients among all eigenvectors are less than 0.3, indicating weak correlations that meet the evaluation requirements.

#### 3.1.3 Driving style clustering based on DE-K-means.

As a classic algorithm in swarm intelligence optimization, Differential Evolution (DE) performs optimization through iterative processes of population initialization, mutation, crossover, recombination, and selection. It relies on collaborative search by multiple agents across a multidimensional solution space and gradually approaches the global optimal solution via algebraic evolution. However, it often suffers from premature convergence when tackling high-dimensional, complex problems. To overcome this limitation, this study combines the global search capabilities of DE with the local search characteristics of K-Means. A two-stage optimization framework is constructed to simultaneously improve the algorithm’s convergence efficiency and stability [28]. The specific steps are as follows:

Step 1: Encoding. Randomly select k cluster centers from the dataset samples and encode them using real-number encoding. This ensures a one-to-one mapping between each feasible solution and its corresponding encoding.
Step 2: Population Initialization. Randomly select k initial cluster centers from the dataset, with each cluster center corresponding to an individual within the algorithm. Repeat this process NP times to construct the initial population.
Step 3: Mutation. Perform mutation operations on the individuals in the population to generate new mutated individuals.
Step 4: Crossover. Conduct crossover operations between the parent individuals and the mutated individuals obtained in Step 3 to produce new candidate individuals.
Step 5: Fitness Evaluation. Calculate the fitness values of the candidate individuals and compare them with those of their parent individuals. Repeat Steps 3–5 iteratively until the maximum number of evolutionary generations set by the algorithm is reached.
Step 6: Clustering. Use the optimal individuals generated by the algorithm as the initial clustering centers for K-Means and initiate subsequent clustering operations.

**Fig 12:**
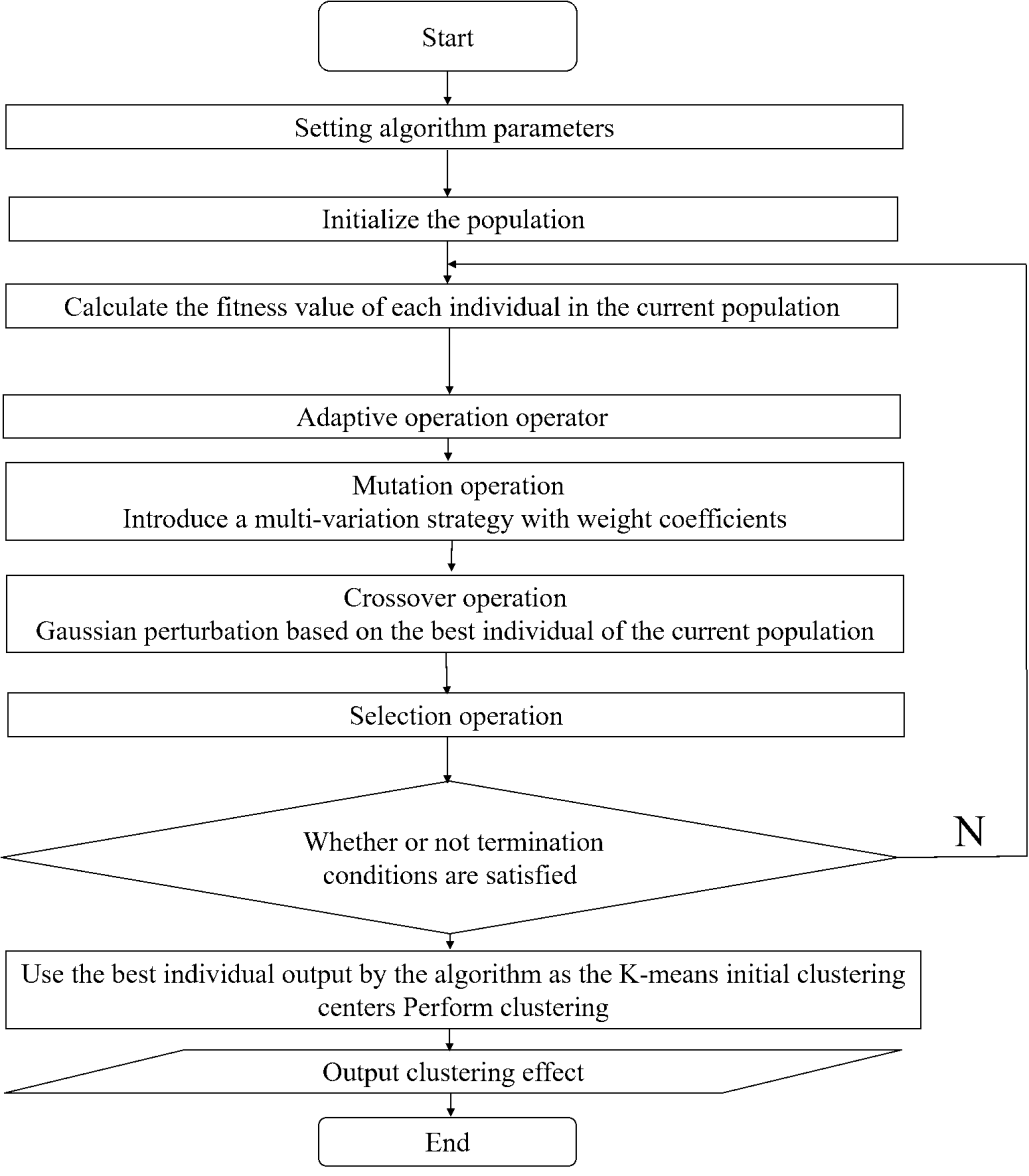
The flow of the K-Means clustering algorithm based on differential evolution.

The algorithm’s workflow is shown in Fig 12.

### 3.2 Construction and calibration method of car-following mode based on driving behavior characteristics behavior

#### 3.2.1 Establishing following distance rules for vehicles on mountainous curved road sections.

The following-vehicle driving model is characterized by the rear driver making acceleration or deceleration decisions based on real-time perception of the leading vehicle’s speed changes and distance. These decisions are guided by the principle of balancing risk perception and efficiency. The primary types and characteristics of the following models are presented in Table 5.

**Table 5:** Main types and characteristics of car-following models.

Model Type	Representative Model	Core Principle	Advantages and Disadvantages
Stimulus-Response Model	GM Model	Rear vehicle acceleration is equal to the sensitivity coefficient multiplied by the ratio of the front vehicle speed difference to the vehicle distance.	This model is simple and easy to use; however, it struggles with parameter calibration and fails to accurately capture complex driving behaviors. behavior
Safe Distance Model	Gipps Model	This model calculates a safe following speed based on maximum braking capacity and reaction time.	It has a clear physical basis and is suitable for low-density traffic, but it does not adequately account for aggressive driving behaviors.
Optimal Velocity Model	OVM Model	Rear vehicle acceleration is proportional to the difference between the current speed and the “optimal speed,” which is based on headway.	This model can reproduce traffic wave propagation, yet it inadequately describes scenarios involving emergency braking.
Intelligent Driving Model (IDM)	IDM Model	This model dynamically adjusts acceleration by integrating vehicle spacing, relative speed, and desired speed.	Its behavior closely mirrors real-world driving, offering strong interpretability of its parameters, though it is computationally expensive.
Cognitive Threshold Model	Psycho-Physical Model	Drivers adjust their speed only when the vehicle spacing falls below a psychological threshold.	This model reflects the discontinuity in human decision-making; however, quantifying these thresholds remains challenging.

In modelling driver-following behavior, accurately capturing the heterogeneity of driving characteristics is crucial to enhancing model applicability. Zhang et al. [29] systematically compared the simulation performance of four classic models. They found that the Intelligent Driver Model (IDM) demonstrated unique advantages across multiple evaluation dimensions. The findings indicate that compared to other models, IDM not only reproduces actual following behavior with higher precision. It also features a more theoretically complete parameter system. Each parameter has a clear physical meaning, such as the desired speed and the safe following distance. This characteristic significantly enhances the model’s ability to account for individual driver differences. The IDM, jointly developed by Helbing D and Hennecke A, has become a classic tool in traffic flow research due to its straightforward structure and broad applicability. The model employs a second-order differential equation as its mathematical framework. It accounts for multiple variables, including vehicle speed, following distance, and the speed difference between the preceding and trailing vehicles. A significant advantage of the IDM is its ability to generate a continuous acceleration function. This output curve maintains smooth transitions across diverse traffic scenarios, demonstrating strong adaptability. The model can be expressed as follows:


$$\begin{array}{c} a_{n}\left(t\right)=a_{\text{0}}\left[1-{\left(\frac{v_{n}\left(t\right)}{{\tilde{v}}_{n}}\right)}^{\text{4}}-{\left(\frac{{\tilde{s}}_{n}\left(t\right)}{s_{n}\left(t\right)}\right)}^{\text{2}}\right] \end{array}$$ (9)

$$\begin{array}{c} {\tilde{s}}_{n}\left(t\right)=s_{\text{0}}+v_{n}\left(t\right)\tilde{T}+\frac{v_{n}\left(t\right)\Delta v\left(t\right)}{2\sqrt{a_{\text{0}}b}} \end{array}$$ (10)

$$\begin{array}{c} \Lambda v\left(t\right)=v_{n-1}\left(t\right)-v_{n}\left(t\right) \end{array}$$ (11)

In the above Equation: an(t) represents the rear vehicle’s acceleration at time t; vn(t) denotes the rear vehicle’s instantaneous velocity at time t; vn−1(t) indicates the front vehicle’s velocity at time t; $\tilde{v}n$ signifies the rear vehicle’s desired velocity; sn(t) represents the inter-vehicle distance between the front and rear vehicles at time t; $\tilde{s}n\left(t\right)$ denotes the rear vehicle’s desired distance; $s_{0}$ signifies the minimum required distance between vehicles; $\tilde{T}$ indicates the rear vehicle’s desired time interval; $a_{0}$ represents the maximum acceleration; b denotes the comfortable deceleration value; Δv(t) signifies the velocity difference between the front and rear vehicles.

Research indicates a significant exponential relationship between curve radius and vehicle operating speed. Specifically, smaller radii not only result in lower average speeds but also induce pronounced speed fluctuations, characterized by substantial deceleration upon entry and vigorous acceleration at the exit [30,31]. Furthermore, to maintain lateral stability during cornering, the required centripetal force must remain within the maximum lateral friction provided by the road surface. According to the theoretical derivations in Chapter 17 of Forensic Engineering Investigation, this mechanical constraint defines the critical threshold for safe cornering speeds [32]. Building upon these findings, the present study refines the existing model by incorporating the curve radius and the critical speed vmax to ensure that the desired speed $v_{desired}$ remains within a physically safe and achievable range (see Eq 15). As the radius decreases, drivers lower their desired speed due to increased perceived risk, which subsequently triggers the deceleration term to reflect risk-avoidance decision-making. Conversely, when the radius exceeds a specific threshold, the perception of geometric constraints diminishes, and drivers tend to treat the curve as a straight segment. This modification mechanism not only accounts for the static constraints imposed by curve geometry on desired speed but also captures the dynamic car-following behavior at curves through the acceleration term. Consequently, the refined model provides a more accurate simulation of vehicle motion patterns under complex alignment conditions. The model can be expressed as follows:


$$\begin{array}{c} a_{n}\left(t\right)=a_{\text{0}}\left[1-{\left(\frac{v_{n}\left(t\right)}{v_{desired}}\right)}^{\text{4}}-{\left(\frac{{\tilde{s}}_{n}\left(t\right)}{s_{n}\left(t\right)}\right)}^{\text{2}}\right] \end{array}$$ (12)

$$\begin{array}{c} {\tilde{s}}_{n}\left(t\right)=s_{\text{0}}+v_{n}\left(t\right)\tilde{T}+\frac{v_{n}\left(t\right)\Delta v\left(t\right)}{2\sqrt{a_{\text{0}}b}} \end{array}$$ (13)

$$\begin{array}{c} \Lambda v\left(t\right)=v_{n-1}\left(t\right)-v_{n}\left(t\right) \end{array}$$ (14)

$$\begin{array}{c} v_{desired}=min\left(v_{max},\sqrt{\mu gR}\right) \end{array}$$ (15)

In the above Equation: an(t) represents the rear vehicle’s acceleration at time t; vn(t) denotes the rear vehicle’s instantaneous velocity at time t; vn−1(t) indicates the front vehicle’s velocity at time t; $v_{desired}$ refers to the desired speed; sn(t) signifies the distance between the two vehicles at time t; $\tilde{s}n\left(t\right)$ represents the desired distance for the rear vehicle; s0 is the minimum required distance between vehicles; $\tilde{T}$ denotes the desired time gap for the rear vehicle; a0 indicates the maximum acceleration; b represents the comfortable deceleration value; Δv(t) signifies the speed difference between the front and rear vehicles; $v_{desired}$ refers again to the desired speed; *R* is the curve radius; *μ* denotes the road surface friction coefficient; *g* indicates gravitational acceleration; vmax represents the maximum driving speed.

#### 3.2.2 Calibration of vehicle following model for mountainous curved road sections.

In transportation system analysis, accurately describing vehicle-following behavior is crucial for understanding traffic flow characteristics. The essence of the parameter calibration process is to achieve a globally optimal configuration of complex system parameters by dynamically linking mathematical modelling and measured data. This technical process can be articulated as follows: Based on the theoretical framework of numerical optimization, a mapping relationship is established between the model parameter space and the observed data space. Iterative optimization algorithms are employed to minimize multidimensional discrepancies between simulation outputs and observed data, ensuring convergence to a predefined threshold.

**Fig 13:**
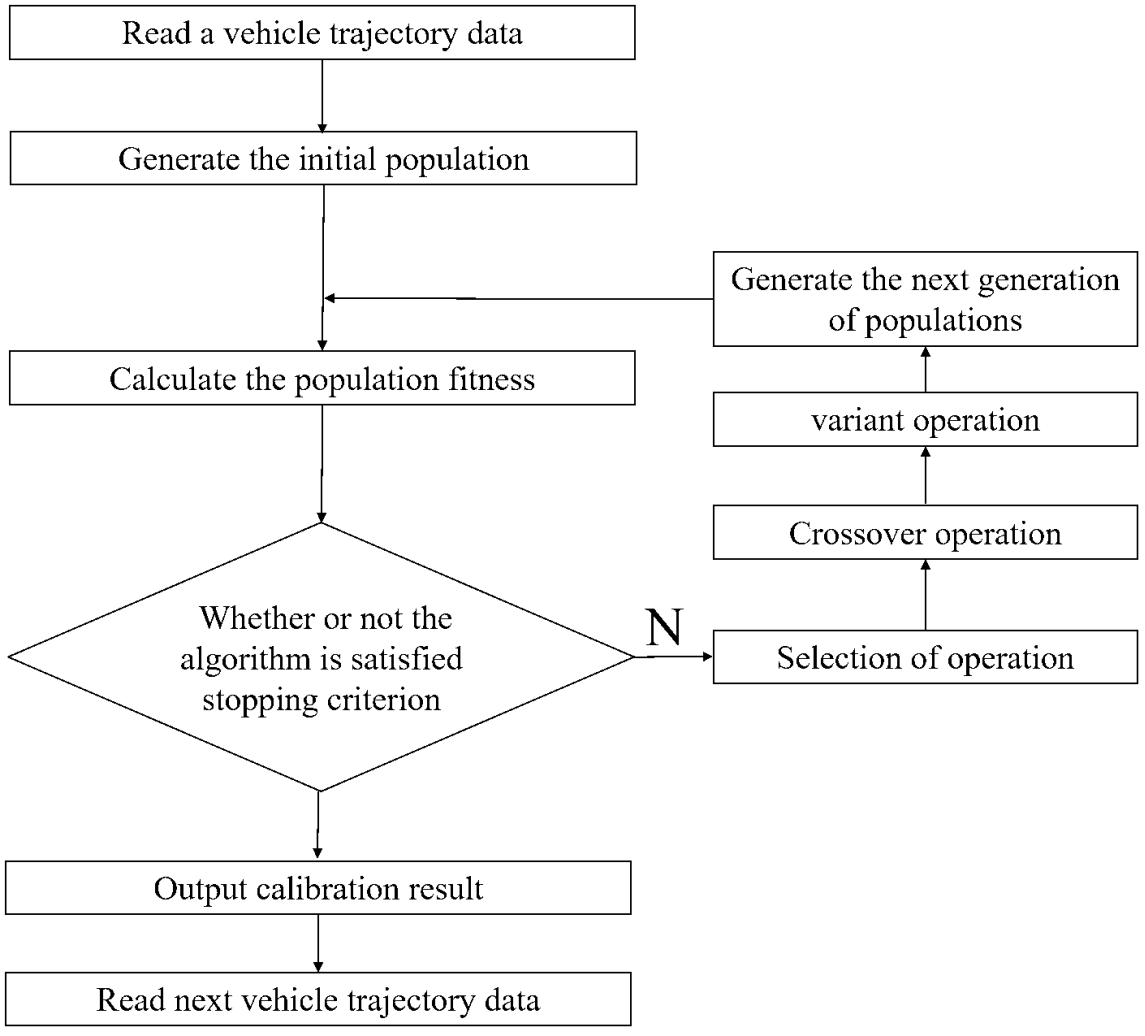
Genetic algorithm calibration of the car-following model parameter process.

The IDM-Chase model is a widely used micro-level traffic flow model. Genetic algorithms, as powerful optimization tools, offer an efficient solution for calibrating the IDM model’s parameters. The core of this algorithm involves continuously generating and evaluating a set of potential solutions through iterative processes. It identifies optimal candidate solutions and applies reproduction and mutation operations, to produce a new generation of solutions. The ultimate goal is to discover the optimal solution to the problem. Therefore, this study employs a genetic algorithm for model parameter calibration, as illustrated in Fig 13.

Based on the analysis of driving behavior characteristics, the extracted event trajectory data undergo further in-depth processing. According to predefined classification labels, these trajectory data are categorized into three distinct types, each corresponding to specific characteristics of driving behavior. To ensure the accuracy and representativeness of subsequent research, data from 10 vehicles are carefully selected from the following trajectory data sets corresponding to each driving behavior characteristic for calibration. A scientifically sound data partitioning strategy is employed for each vehicle’s subsequent trajectory. The first 80% of each trajectory is designated as the training set, enabling the model to learn intrinsic patterns in various driving behavior characteristics through extensive feature analysis. The remaining 20% is reserved as the validation set to test the trained model. Validation set testing evaluates the model’s performance on untrained data, which assesses its generalization capability and accuracy in recognizing different driving behavior characteristics. This approach ensures the scientific rigor and reliability of the entire research process.

In practical modelling and analysis, calibration is essential to ensure models accurately reflect real-world conditions. Genetic algorithms, as powerful optimization tools, offer significant advantages for model calibration. MATLAB provides a feature-rich Genetic Algorithms Toolbox that enables efficient calibration. When utilizing this toolbox, the appropriate configuration of algorithm control variables is crucial for achieving calibration results that closely align with actual conditions. These control variable settings significantly influence both the algorithm’s performance and the final calibration outcomes. In the toolbox, the configuration of algorithm control variables for parameter calibration is presented in Table 6, while the boundary settings are detailed in Table 7.

**Table 6:** Genetic algorithm calibration Settings.

Operating Parameters	Settings
Selection Operator	Roulette Wheel Selection
Crossover Probability	0.8
Mutation Probability	0.2
Population Size	200
Iteration Count	200
Selection Operator	Roulette Wheel Selection

**Table 7:** Bounding settings.

Calibration Parameters	Definition	Boundary Range
s0 (m)	Minimum Headway	[0, 50]
$\tilde{T}$ (s)	Desired Time Gap	[0.1, 10]
$a_{0}$ (m/s^2^)	Maximum Acceleration	[0, 5]
$\tilde{v}n$ (m/s)	Desired Speed	[1, 100]
*b* (m/s^2^)	Comfortable Deceleration	[0, 5]

## 4 Results and discussion

### 4.1 Clustering analysis of driving styles based on DE-K-Means

#### 4.1.1 Analysis of clustering results.

This study refers to Xu et al. [33] regarding the impact of inherent driving styles on traffic risk prediction. Their study indicated that different drivers exhibit distinct inherent driving styles, and incorporating driving behavior characteristics into the risk analysis framework enhances the model’s explanatory power for driving behavior heterogeneity. The results demonstrated that integrating inherent driving styles into the model reduced prediction errors by 3.9% and facilitated more targeted decision-making. This underscores the necessity and rationality of classifying driving behavior characteristics in car-following modeling. Furthermore, by comparing the clustering performance under different numbers of clusters, they ultimately set the number of clusters to three and classified driving styles into three categories: Aggressive, Normal, and Cautious. Their study provides a reference basis for classifying driving behavior characteristics into three categories in this paper.

**Fig 14:**
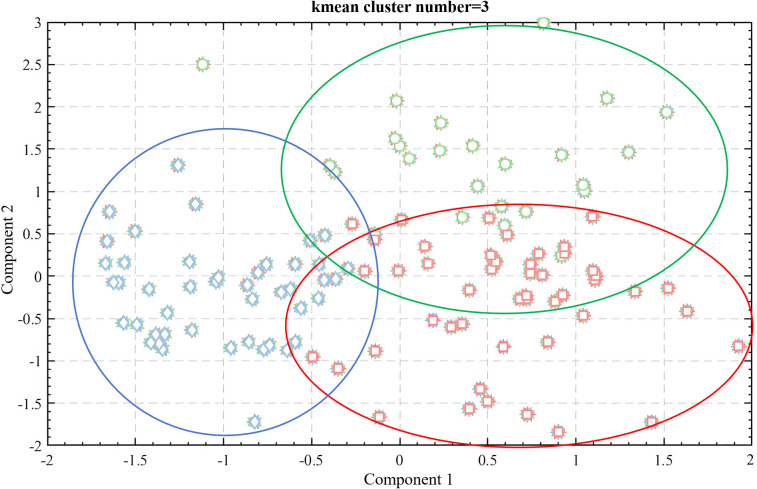
Clustering result analysis diagram.

Based on this, this study employs a Differential Evolution-based K-Means clustering algorithm to perform cluster analysis on four common factors, namely lateral movement factor, safety distance factor, longitudinal movement factor, and acceleration behavior factor. By comprehensively considering clustering evaluation metrics, sample distribution, and the interpretability of driving behavior characteristics, the number of clusters is ultimately determined as three. The clustering results are shown in Fig 14.

After clustering, the sample size and proportion of each of the three driving style categories were calculated, with the detailed statistical results presented in Table 8. Specifically, Cluster 1 contains 50 samples, accounting for 21.18% of the total dataset; Cluster 2 contains 90 samples, accounting for 38.14%; and Cluster 3 contains 96 samples, accounting for 40.68%. The total dataset comprises 236 samples. All three clusters have sufficient sample sizes, and no extreme imbalance issue with excessively small sample size in any single cluster is observed, which validates the rationality of the clustering scheme from the perspective of sample distribution.

**Table 8:** Sample size and proportion distribution of the three driving style clusters.

Cluster	Sample size	Proportion
Cluster 1	50	21.18%
Cluster 2	90	38.14%
Cluster 3	96	40.68%
Total	236	100%

To validate the performance of the differential evolution-based K-Means clustering algorithm, its clustering results were compared with those of K-means and GMM. This comparison is detailed in Table 9. Among these algorithms, the differential evolution-based K-Means demonstrated superior clustering performance.

**Table 9:** Performance index difference of clustering algorithm.

Performance Index	DE-GMM	DE-K-Means	GMM-K-Means
MAE	0.4153	0.9915	1.0339
MSE	0.7542	1.1949	1.3559

**Fig 15:**
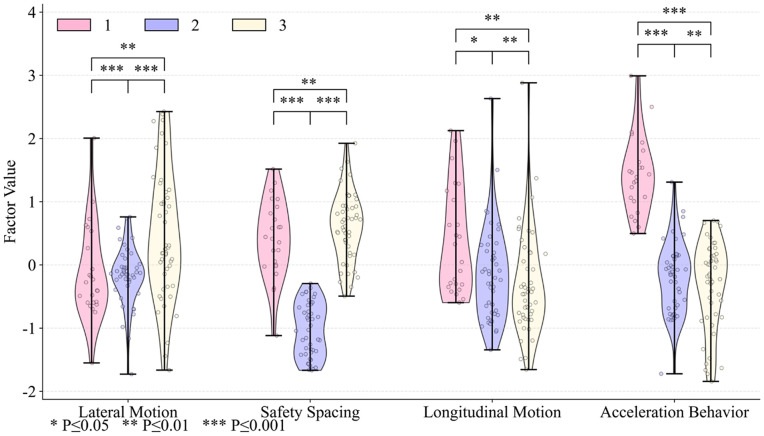
Intergroup differences and significance test results of driving behavior characteristics among the three driving styles.

To verify the scientific validity of the clustering scheme, this paper conducts an inter-group significance test on four common factors across the three driving behavior clusters, namely the lateral motion factor, safety spacing factor, longitudinal motion factor, and acceleration behavior factor, as shown in Fig 15. The statistical results indicate that all clustered groups differ significantly in the above parameters. In particular, the safety spacing factor and acceleration behavior factor reach a highly significant level (P < 0.001), revealing distinct differences in driving risk perception among drivers with different driving styles. The violin plot, combined with the scatter distribution, further intuitively demonstrates the density and overlap of factor scores. It is verified that the clustering results effectively capture the heterogeneity in driving behavior on mountainous curved sections, providing a rigorous statistical basis for the subsequent targeted analysis of the traffic safety characteristics of each group.

#### 4.1.2 Classification of driving behavior characteristics.

**Fig 16:**
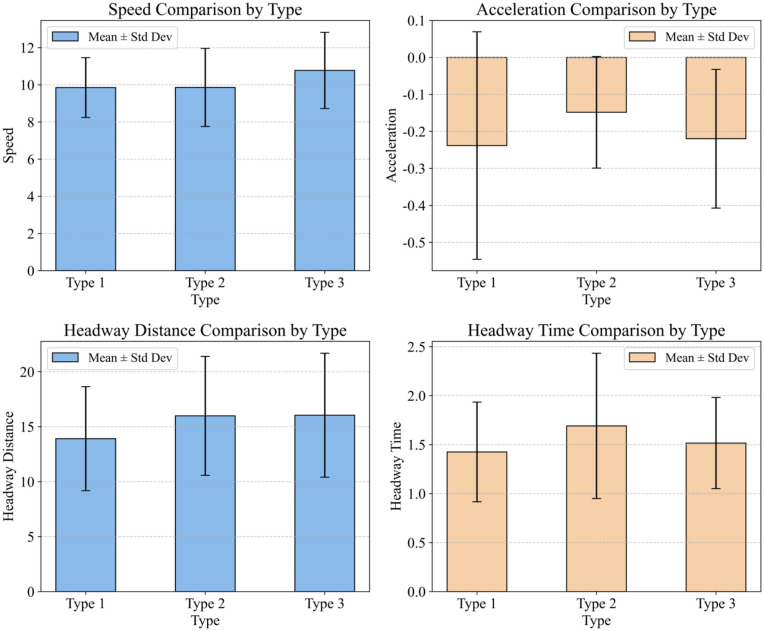
Differences in Driver Attributes Across Categories.

After completing the clustering of drivers’ behavioral characteristics, key driving metrics such as average speed, peak speed, peak acceleration, and average headway were analyzed for the three distinct driver categories. This analysis, as illustrated in Fig 16, allows for clear differentiation of driving behaviors among the three groups. The study results indicate significant differences in these critical driving parameters across different driver types.

**Fig 17:**
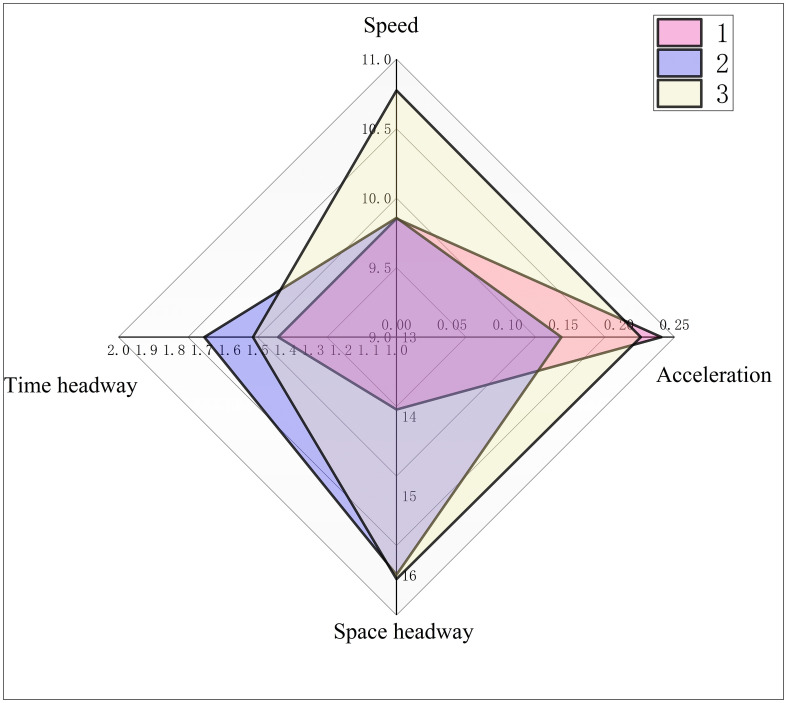
Parameter radar chart.

This study explores driving behavior characteristics using multidimensional data visualization. Fig 17 uses a radar chart to show the mean distribution patterns of various parameter indicators. Based on clustering analysis results, Fig 14 systematically identifies the parameter combinations of three typical driving patterns. The first group shows a high average speed, a minimal following distance, and significant peak rates of change in longitudinal acceleration. This aggressive driving style can improve individual vehicle efficiency, but significantly increases the risk of rear-end collisions and skidding in curves. The second group maintains the greatest distance from preceding vehicles and exhibits minimal, smooth changes in acceleration, reflecting conservative driving traits. Their excessive caution improves safety but may hinder overall traffic flow efficiency. The third group presents moderate characteristics across all metrics, representing a balanced standard driving mode that optimizes both safety and efficiency. This mode can act as a benchmark for traffic management and intelligent driving systems. Based on behavioral trait spectra and their potential impacts on safety and efficiency, the study classifies the three driving patterns as aggressive, conservative, and standard.

#### 4.1.3 Analysis of characteristics of different driving behaviors.

**Fig 18:**
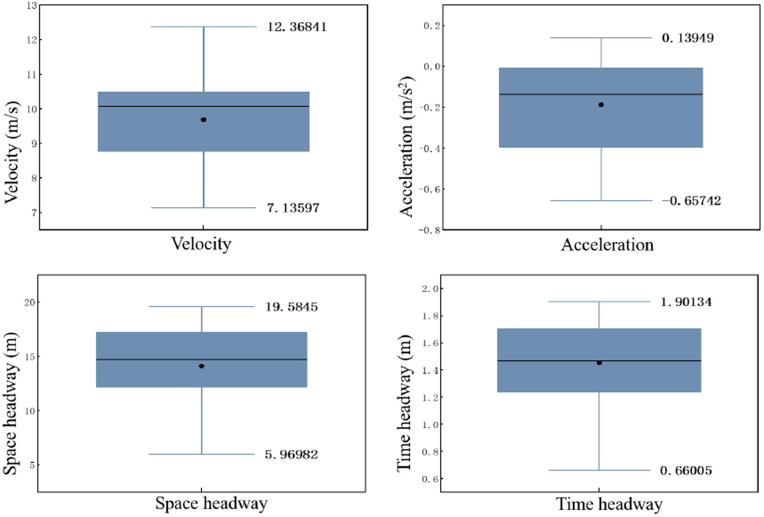
Analysis of aggressive driver characteristics.

To provide a more intuitive illustration of vehicle operation characteristics across aggressive, standard, and conservative drivers, box plots were constructed for each driver type. These plots depict metrics such as speed, acceleration, headway distance, and headway time distance, as shown in Figs 18–Fig 20.

**Fig 19:**
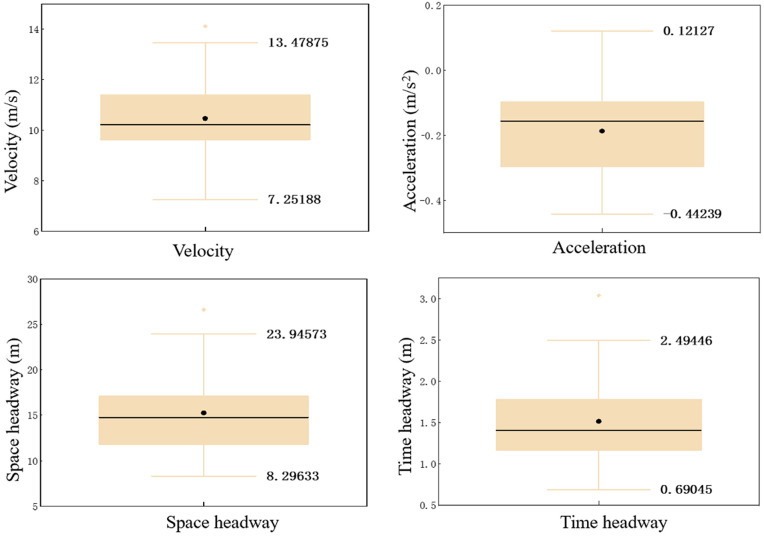
Characteristic analysis of the standard driver.

**Fig 20:**
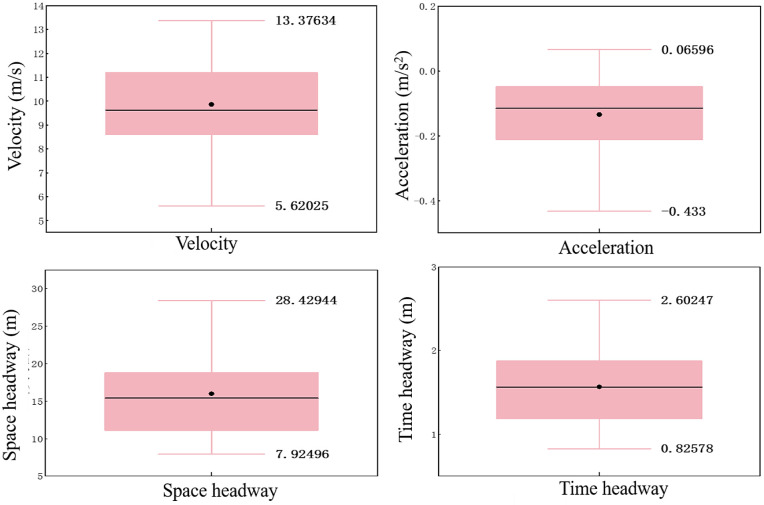
Conservative driver characteristics analysis.

(1) Speed Characteristic Analysis

For aggressive drivers, the median speed box plot is significantly higher than the other two groups. Conservative drivers exhibit the lowest median speed, with driving speeds concentrated in the lower range, reflecting their cautious driving behavior. Standard drivers exhibit a speed distribution across both extremes, indicating typical driving behavior.

(2) Acceleration Characteristic Analysis

Comparing the acceleration distribution charts of the three driver types reveals that the box plot for aggressive drivers shows a wide distribution, with significantly higher positive and negative acceleration extremes than those of the other groups, indicating frequent abrupt acceleration and deceleration. They exhibit the highest number of outliers, reflecting unpredictable driving behavior. In contrast, the acceleration distribution for conservative drivers is highly concentrated in the low-absolute-value range, reflecting smooth driving habits. Standard drivers exhibit moderate dispersion in the acceleration distribution, with few outliers, consistent with conventional driving behavior.

(3) Analysis of Front-to-Front Distance and Front-to-Front Time Gap

For aggressive drivers, both front-to-front distance and front-to-front time gap exhibit the smallest median values, indicating short following distances with substantial variability and greater safety risks. Conversely, conservative drivers exhibit the highest median values for both distances, maintaining stable following distances that meet safety standards, with no tailgating outliers, reflecting sound driving awareness. For standard drivers, the following distances are centrally distributed with a median close to the traffic regulation recommendation, reflecting conventional safety consciousness.

**Fig 21:**
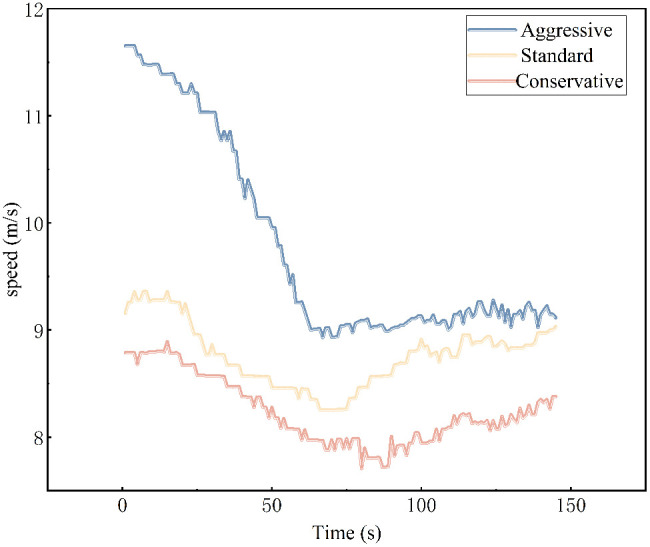
Speed variation characteristics of different driving behaviors.

To more intuitively compare differences among drivers with distinct driving-behaviour characteristics, one trajectory was selected from each category. The speed variation patterns for different driving behaviors are illustrated in Fig 21 below.

Analysis of Fig 21 reveals that drivers with distinct behavioral characteristics exhibit different patterns of speed variation when navigating mountainous road curves. Overall speed trends show an initial increase, followed by a period of fluctuation, and then another rise. This pattern reflects significant differences among driver types. Aggressive drivers exhibit markedly greater speed variability than conservative and standard drivers. Aggressive drivers exhibit consistently higher, more concentrated speed values and extremely volatile speed fluctuations. This contrasts sharply with the relatively stable speed changes observed among conservative drivers and the moderate speed variations observed among standard drivers. Aggressive drivers start time at the highest initial speed, approaching 12 m/s, which then decreases rapidly before settling into minor fluctuations. Standard drivers start at lower speeds than aggressive drivers, experience a brief initial rise followed by a sustained decline, and later fluctuate between 8.5–9 m/s, exhibiting more minor overall variations than aggressive drivers. Conservative drivers begin at the lowest initial speed, maintain a gradual downward trend throughout, and stabilize at around 8 m/s, showing the smallest fluctuations. Aggressive drivers exhibit the highest initial speeds but experience a significant decline, whereas conservative drivers maintain the most stable overall speeds. Standard drivers show intermediate variations between these two extremes. This reflects distinct strategic differences in speed adaptation across the three driving styles, indicating a correlation between speed distribution and evolving driving characteristics.

### 4.2 Construction and analysis of the following model based on driving behavior characteristics

#### 4.2.1 Calibration and analysis of car-following models for vehicle operation on mountainous curved road sections.

When investigating the relationship between driving behavior and rule-following behavior, calibration is essential given the diversity of driving behaviors. This ensures accurate identification of different manifestations of driving behavior in follow-up scenarios. We calibrated three types of typical driving behavior according to established rules. Given the random nature of genetic algorithms, which yield varying optimization results, 40 calibration operations were conducted for each driver type’s trajectory data to determine the optimal solution. Post-calibration, we established screening criteria based on empirical data to eliminate outliers. After extensive testing, data collection, and rigorous analysis, the calibrated results for each driver type, including mean, standard deviation, maximum, and minimum values, were finalized. Specific data are detailed in Table 10.

**Table 10:** Calibration results.

Segment	Parameter	Description	Result	
			Mean	Standard Deviation
Aggressive	$s_{0}$ (m)	Minimum Headway	11.7461	3.1618
	$\tilde{T}$ (s)	Desired Time Gap	1.3025	1.3025
	$a_{0}$ (m/s^2^)	Maximum Acceleration	0.1774	0.1774
	*b* (m/s^2^)	Comfort Deceleration	0.2025	0.2025
	$a_{0}$ (m/s)	Desired Speed	11.3017	11.3017
Standard	$s_{0}$ (m)	Minimum Headway	14.1128	5.6912
	$\tilde{T}$ (s)	Desired Time Gap	1.4995	1.4995
	$a_{0}$ (m/s^2^)	Maximum Acceleration	0.1288	0.1288
	*b* (m/s^2^)	Comfort Deceleration	−0.1809	−0.1809
	$\tilde{v}n$ (m/s)	Desired Speed	9.5387	9.5387
Conservative	$s_{0}$ (m)	Minimum Headway	15.8683	5.4909
	$\tilde{T}$ (s)	Desired Time Gap	1.5691	1.5691
	$a_{0}$ (m/s^2^)	Maximum Acceleration	0.0761	0.0761
	*b* (m/s^2^)	Comfort Deceleration	0.1574	0.1574
	$\tilde{v}n$ (m/s)	Desired Speed	9.5352	9.5352
	$s_{0}$ (m)	Minimum Headway	11.7461	3.1618

The calibrated data reveal significant differences among aggressive, standard, and conservative drivers across multiple key parameters. For instance, the average maximum acceleration for aggressive drivers is 0.1774 m/s^2^, which is markedly higher than that of standard and conservative drivers. Standard drivers exhibit a moderate average maximum acceleration of 0.1288 m/s^2^, aligning with typical acceleration demands in conventional driving scenarios. In contrast, conservative drivers exhibit the lowest average maximum acceleration 0.0761 m/s^2^. It is important to note that the maximum acceleration parameters calibrated in this study range from 0.0761 to 0.1774 m/s^2^, which are generally lower than the commonly accepted values for straight road sections. This discrepancy arises from the unique driving conditions on mountainous curves. First, from a vehicle dynamics perspective, cornering imposes constraints on centripetal acceleration. Drivers must allocate part of their longitudinal acceleration capacity to maintain lateral stability, thus limiting pure longitudinal acceleration. Second, from a driving behavior perspective, the curved road layout restricts visibility. To mitigate potential risks, such as oncoming vehicles or roadside obstacles, drivers adopt more cautious strategies and actively avoid abrupt acceleration or deceleration maneuvers. Therefore, the calibrated lower acceleration values do not indicate model bias; instead, they reflect the common behavioral characteristic of drivers who prioritize stability and actively suppress aggressive cornering. This finding indirectly validates the model’s ability to capture real-world driving constraints on mountainous roads.

Aggressive drivers often underestimate driving risks and overestimate their skills and reaction times. They believe they can react quickly enough to avoid collisions, even when closely following the vehicle ahead, leading them to maintain a smaller minimum safe following distance. Eager to overtake other vehicles or quickly navigate through road sections, they frequently accelerate to reach higher speeds for rapid passing or forward movement. In contrast, conservative drivers have a clear understanding of potential driving risks. They recognize that, even when adhering to proper driving practices, they may still be influenced by other vehicles or unexpected situations. To ensure adequate time and space to respond in any scenario, they maintain a larger minimum safe following distance, thereby reducing the likelihood of collisions. Further in-depth research that combines these parameter differences with classifications of actual driving behavior reveals a strong intrinsic connection between them. This connection effectively supports the rationale for classifying drivers based on their behavioral characteristics.

#### 4.2.2 Verification and analysis of model results.

(1) Macroscopic error verification

During the driving model calibration, a diverse array of performance metrics is available for selection. This paper identifies vehicle position and velocity as the chosen performance metrics. The goodness-of-fit function primarily assesses the discrepancy between the model’s calculated results and the actual performance metrics. By calculating the errors between simulations of various driving behavior characteristics and the actual data, the root mean square error (RMSE) metric is derived to evaluate the calibrated model. The expression for the error metric is as follows:


$$\begin{array}{c} S_{RMSE}=\sqrt{\frac{\sum_{i=1}^{N}(\overset{sim}{\underset{i}{S}}-\overset{abs}{\underset{i}{S}}{)}^{\text{2}}}{N}} \end{array}$$ (16)

In the formula, *i* denotes the index, $\overset{sim}{\underset{i}{S}}$ is the *i*-th simulated value, $\overset{abs}{\underset{i}{S}}$ is the *i*-th actual value, and *N* is the total count.

This study uses numerical simulation for validation. Vehicle position and velocity are derived from vehicle trajectory data in the validation dataset, which includes drivers exhibiting various driving behaviors. The validation error is calculated, as shown in Table 11.

**Table 11:** Validation error.

Driving behavior Characteristics	Parameter	Mean	Standard Deviation	Maximum
Aggressive	Location	1.5436	2.5927	6.5694
	Velocity	0.8523	0.8531	2.6190
Standard	Location	0.7218	0.7552	2.7532
	Velocity	0.9397	1.0010	3.4194
Conservative	Location	0.5622	0.6290	1.7817
	Velocity	0.7222	0.3580	1.6260

Overall, conservative driving behavior characteristics exhibit superior stability and accuracy, as measured by root-mean-square error (RMSE), for both position and velocity. Lower average values, smaller standard deviations, and reduced differences between maximum and minimum values evidence this. To quantitatively evaluate the performance gap between the integrated IDM model, which combines driving behavior and road geometry, and the original model, this study conducted a statistical analysis of micro-trajectory prediction capabilities using RMSE, as presented in Table 12. The table shows that the IDM model integrating road geometry and driving behavior characteristics has a smaller average RMSE, indicating excellent calibration performance.

**Table 12:** Comparison of RMSE.

Driving Behavior Characteristics	Model	RMSE Average	
		Location	Velocity
Aggressive	Original IDM	1.7246	1.4961
	Integrated IDM	1.6134	0.8523
Standard	Original IDM	1.1867	1.0487
	Integrated IDM	0.7218	0.9397
Conservative	Original IDM	0.9787	0.9371
	Integrated IDM	0.5622	0.7222

(2) Microscopic Trajectory Verification

**Fig 22:**
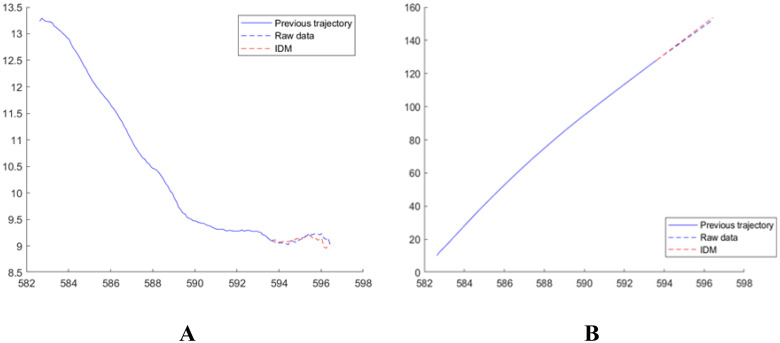
Comparison chart of radical simulation and actual situation. A: Speed indicator; **B:** Position indicator.

For this experiment, the top 80% of the trajectory data was selected to calibrate the IDM model, while the remaining 20% was reserved for model validation. To assess the effectiveness of the following-distance rules at a micro level, one trajectory from each driver type was extracted for validation. The results, presented in Figs 22–Fig 24, indicate that the calibrated IDM model closely matches the validated trajectories.

**Fig 23:**
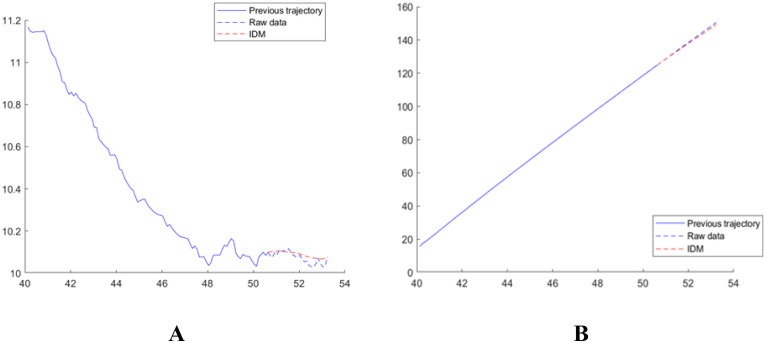
Comparison chart of standard simulation and actual situation. A: Speed indicator; **B:** Position indicator.

**Fig 24:**
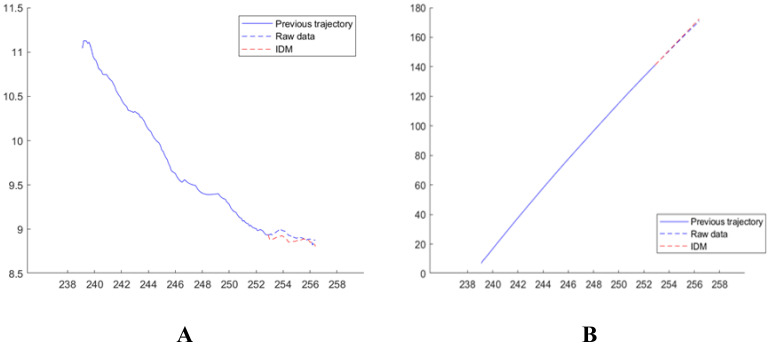
Comparison chart of simulation and actual comparison. **A**: Speed indicator; **B:** Position indicator.

In the rear vehicle position curve, the solid blue line represents the first 80% of actual position data used for model calibration. The dashed blue line indicates the final 20% of actual position data for validation. The dashed red line shows the simulated positions from the calibrated IDM model over the last 20% of the time interval, used to assess the accuracy of position predictions. In the rear vehicle speed curve, the solid blue line corresponds to the first 80% of the actual speed data used for calibration, while the dashed blue line represents the final 20% used for validation. The dashed red line shows the model’s simulated speed over the last 20% of the time interval, used to evaluate its speed-prediction capability.

Simulation results indicate that all three driver types exhibit a decreasing speed trend during cornering. The aggressive driver type shows the most significant speed fluctuations, while the conservative type shows the least, with the standard type falling in between—consistent with their clustering characteristics. High-frequency fluctuations in real-time speed modulation in the trajectory data are smoothed out in the model predictions. Given the relatively short duration of the validation trajectory, the simulation effectively captures micro-level position and speed trajectories. This difference arises from the need for drivers to adjust their longitudinal speed dynamically in real time to accommodate changes in road geometry as curve curvature varies.

## 5 Conclusions

Car-following behavior modeling on curved sections of mountainous highways is crucial for improving traffic safety and operational efficiency. Although traditional car-following models have laid a foundation for vehicle longitudinal control, they often fail to accurately characterize actual driving dynamics due to the significant heterogeneity of driving behavior and complex geometric alignment constraints in mountainous terrain. This study proposes a method for characterization that couples driving-style classification with road-curve radius to enhance the characterization accuracy of the Intelligent Driver Model (IDM) in mountainous environments. The findings are highly consistent with the systematic conclusion of Yao et al. [34] regarding the influence of car-following heterogeneity on traffic safety and sustainability.

The results reveal that coupling driving behavioral characteristics significantly improves the model’s descriptive capability. By adopting the differential optimized evolution-optimized K-Means clustering algorithm, the clustering-based classification method enables the model to capture significant differences in speed, acceleration, and vehicle spacing among various driving styles more precisely than conventional unclassified car-following models. The improved IDM model, incorporating the curve radius correction factor and style characteristic parameters, outperforms the traditional model in reconstructing real car-following trajectories and achieves higher accuracy, particularly in capturing speed fluctuations within curves and maintaining safe vehicle spacing.

Although drone-based trajectory data curves. Current research is primarily limited to typical two-lane horizontal-curve sections in mountainous areas with good sight distance and dry pavement conditions. Future research will collect data conditions across combinations of different curve radii and longitudinal slopes, as well as various weather conditions, to explore the car-following mechanism in multi-scenario environments. Meanwhile, integrating the proposed model with conflict risk prediction modelling [35] will further elucidate the evolutionary logic of vehicle car-following risk in mountainous curved environments.

This study of driving-behavior heterogeneity with road geometric features provides an effective framework for car-following modelling on mountainous curves. The findings lay a foundation for and offer new perspectives for modelling traffic safety management on mountainous highways and offer new perspectives on modelling continuous multi-curve sections, real-time conflict risk prediction, and the design of autonomous driving car-following strategies in mountainous areas.

## Abbreviations

DE: differential evolution
GA: genetic algorithm
ANN: artificial neural network
IDM: intelligent driving model
RMSE: root mean square error
GMM: Gaussian mixture model
KCF: Kernelized correlation filter.
