# Supplementary material for: A study on the car-following model for mountainous curves incorporating driving behavior characteristics
Source: PLoS One. 2026 Jul 15;21(7):e0352855. doi: 10.1371/journal.pone.0352855 (PMC13372160; doi:10.1371/journal.pone.0352855)
Supplement: Attachment — Submitted filename: The Third Response to Reviewers.docx [file pone.0352855.s002.docx]

Dear editors:

Thank you for your time and effort in reviewing and improving our manuscript entitled "A Study on the Car-Following Model for Mountainous Curves Incorporating Driving Behavior Characteristics". We have carefully considered your comments and suggestions and are now submitting a revised version of the manuscript for further consideration by the journal. In this revision, we have removed the trademarks involved in Figure 4 as per your comments. In the response letter, blue text indicates our explanations, black text represents your original comments, and red text highlights the changes made in the manuscript. We look forward to receiving your assessment and suggestions.

Yours sincerely,

Longjiao Zhang.

Yunnan Yunling Highway Science and Technology Co., Ltd. Kunming 650000, Yunnan, China.

Email: zljyyhs@outlook.com

***Response to the third round of reviewers' comments***

***Thank you for your further review of our second revised manuscript. We now respond point by point to the new comments raised in this round.***

***Comment 1:***

***Regarding Figure 4, please blur or remove any trademarks or copyrighted logos visible in the photographs.***

**Response 1:**

Thank you very much for your valuable comments. We have strictly followed your request and removed all visible trademarks and copyrighted logos from the photographs. The revised figures have been updated in the manuscript, as shown below.

*In addition, as shown in Figure 4, to verify data accuracy, a mechanical measuring wheel (Deli, 0304) and a mobile radar speed gun (Bushnell, 01911) were used as auxiliary data-collection devices. The mechanical measuring wheel can measure the distance between any two observation points captured by the drone, with a measurement accuracy of ±0.5%; the radar speed gun can detect vehicle speed with a measurement accuracy of ±1.0 MPH to ±2.0 KPH.*

*A	B*

*Fig 4. Auxiliary measuring instruments. A: Mechanical distance measuring wheel; B: Mobile radar speed gun.*
