# Supplementary material for: A study on the car-following model for mountainous curves incorporating driving behavior characteristics
Source: PLoS One. 2026 Jul 15;21(7):e0352855. doi: 10.1371/journal.pone.0352855 (PMC13372160; doi:10.1371/journal.pone.0352855)
Supplement: Peer review history

# Peer review: Decision Letter 0

**DOI:** 10.1371/journal.pone.0352855.r001

**Authors:** Zhihong (Arry) Yao

2 Apr 2026

PONE-D-26-06596

A Study on the Car-Following Model for Mountainous Curves Incorporating Driving Behaviour Characteristics

PLOS One

Dear Dr. Zhang,

Thank you for submitting your manuscript to PLOS ONE. After careful consideration, we feel that it has merit but does not fully meet PLOS ONE’s publication criteria as it currently stands. Therefore, we invite you to submit a revised version of the manuscript that addresses the points raised during the review process.

Please submit your revised manuscript by May 17 2026 11:59PM. If you will need more time than this to complete your revisions, please reply to this message or contact the journal office at plosone@plos.org. When you're ready to submit your revision, log on to https://www.editorialmanager.com/pone/ and select the 'Submissions Needing Revision' folder to locate your manuscript file.

Please include the following items when submitting your revised manuscript:

- A letter that responds to each point raised by the academic editor and reviewer(s). You should upload this letter as a separate file labeled 'Response to Reviewers'.
- A marked-up copy of your manuscript that highlights changes made to the original version. You should upload this as a separate file labeled 'Revised Manuscript with Track Changes'.
- An unmarked version of your revised paper without tracked changes. You should upload this as a separate file labeled 'Manuscript'.

If you would like to make changes to your financial disclosure, please include your updated statement in your cover letter. Guidelines for resubmitting your figure files are available below the reviewer comments at the end of this letter.

If applicable, we recommend that you deposit your laboratory protocols in protocols.io to enhance the reproducibility of your results. Protocols.io assigns your protocol its own identifier (DOI) so that it can be cited independently in the future. For instructions see: https://journals.plos.org/plosone/s/submission-guidelines#loc-laboratory-protocols. Additionally, PLOS ONE offers an option for publishing peer-reviewed Lab Protocol articles, which describe protocols hosted on protocols.io. Read more information on sharing protocols at https://plos.org/protocols?utm_medium=editorial-email&utm_source=authorletters&utm_campaign=protocols.

As the corresponding author, your ORCID iD is verified in the submission system and will appear in the published article. PLOS supports the use of ORCID, and we encourage all coauthors to register for an ORCID iD and use it as well. Please encourage your coauthors to verify their ORCID iD within the submission system before final acceptance, as unverified ORCID iDs will not appear in the published article. *Only*  the individual author can complete the verification step; PLOS staff *cannot*  verify ORCID iDs on behalf of authors.

We look forward to receiving your revised manuscript.

Kind regards,

Zhihong (Arry) Yao, Ph.D.

Academic Editor

PLOS One

**Journal Requirements:**

1. When submitting your revision, we need you to address these additional requirements.

Please ensure that your manuscript meets PLOS ONE's style requirements, including those for file naming. The PLOS ONE style templates can be found at

https://journals.plos.org/plosone/s/file?id=wjVg/PLOSOne_formatting_sample_main_body.pdf and

https://journals.plos.org/plosone/s/file?id=ba62/PLOSOne_formatting_sample_title_authors_affiliations.pdf

2. In your Methods section, please include additional information about your dataset and ensure that you have included a statement specifying whether the collection and analysis method complied with the terms and conditions for the source of the data.

3. In your Methods section, please provide additional information regarding the permits you obtained for the work. Please ensure you have included the full name of the authority that approved the field site access and, if no permits were required, a brief statement explaining why.

4. Please note that PLOS One has specific guidelines on code sharing for submissions in which author-generated code underpins the findings in the manuscript. In these cases, we expect all author-generated code to be made available without restrictions upon publication of the work. Please review our guidelines at https://journals.plos.org/plosone/s/materials-and-software-sharing#loc-sharing-code and ensure that your code is shared in a way that follows best practice and facilitates reproducibility and reuse.

5. Thank you for stating in your Funding Statement:

This work was supported by the Science and Technology Innovation and Demonstration Project of Department of Transport of Yunnan Province (No.2023-83(1)), and Project Funding by Yunnan Traffic Science Research Institute Co., Ltd (Nos. JKYZLX-2023-12, JKYZLX-2025-09 and JKYZLX-2025-39).

Please provide an amended statement that declares *all* the funding or sources of support (whether external or internal to your organization) received during this study, as detailed online in our guide for authors at http://journals.plos.org/plosone/s/submit-now.  Please also include the statement “There was no additional external funding received for this study.” in your updated Funding Statement.

Please include your amended Funding Statement within your cover letter. We will change the online submission form on your behalf.

6. Thank you for stating the following financial disclosure:

This work was supported by the Science and Technology Innovation and Demonstration Project of Department of Transport of Yunnan Province (No.2023-83(1)), and Project Funding by Yunnan Traffic Science Research Institute Co., Ltd (Nos. JKYZLX-2023-12, JKYZLX-2025-09 and JKYZLX-2025-39).

Please state what role the funders took in the study.  If the funders had no role, please state: "The funders had no role in study design, data collection and analysis, decision to publish, or preparation of the manuscript."

If this statement is not correct you must amend it as needed.

Please include this amended Role of Funder statement in your cover letter; we will change the online submission form on your behalf.

7. We note that you have indicated that there are restrictions to data sharing for this study. PLOS only allows data to be available upon request if there are legal or ethical restrictions on sharing data publicly. For more information on unacceptable data access restrictions, please see http://journals.plos.org/plosone/s/data-availability#loc-unacceptable-data-access-restrictions.

Before we proceed with your manuscript, please address the following prompts:

a) If there are ethical or legal restrictions on sharing a de-identified data set, please explain them in detail (e.g., data contain potentially identifying or sensitive patient information, data are owned by a third-party organization, etc.) and who has imposed them (e.g., a Research Ethics Committee or Institutional Review Board, etc.). Please also provide contact information for a data access committee, ethics committee, or other institutional body to which data requests may be sent.

b) If there are no restrictions, please upload the minimal anonymized data set necessary to replicate your study findings to a stable, public repository and provide us with the relevant URLs, DOIs, or accession numbers. For a list of recommended repositories, please see

https://journals.plos.org/plosone/s/recommended-repositories. You also have the option of uploading the data as Supporting Information files, but we would recommend depositing data directly to a data repository if possible.

We will update your Data Availability statement on your behalf to reflect the information you provide.

8. PLOS requires an ORCID iD for the corresponding author in Editorial Manager on papers submitted after December 6th, 2016. Please ensure that you have an ORCID iD and that it is validated in Editorial Manager. To do this, go to ‘Update my Information’ (in the upper left-hand corner of the main menu), and click on the Fetch/Validate link next to the ORCID field. This will take you to the ORCID site and allow you to create a new iD or authenticate a pre-existing iD in Editorial Manager.

9. We note that Figures 1, 2 and 3 in your submission contain map/satellite images which may be copyrighted. All PLOS content is published under the Creative Commons Attribution License (CC BY 4.0), which means that the manuscript, images, and Supporting Information files will be freely available online, and any third party is permitted to access, download, copy, distribute, and use these materials in any way, even commercially, with proper attribution. For these reasons, we cannot publish previously copyrighted maps or satellite images created using proprietary data, such as Google software (Google Maps, Street View, and Earth). For more information, see our copyright guidelines: http://journals.plos.org/plosone/s/licenses-and-copyright.

We require you to either present written permission from the copyright holder to publish these figures specifically under the CC BY 4.0 license, or remove the figures from your submission:

a. You may seek permission from the original copyright holder of Figures 1, 2 and 3 to publish the content specifically under the CC BY 4.0 license.

We recommend that you contact the original copyright holder with the Content Permission Form (http://journals.plos.org/plosone/s/file?id=7c09/content-permission-form.pdf) and the following text:

“I request permission for the open-access journal PLOS ONE to publish XXX under the Creative Commons Attribution License (CCAL) CC BY 4.0 (http://creativecommons.org/licenses/by/4.0/). Please be aware that this license allows unrestricted use and distribution, even commercially, by third parties. Please reply and provide explicit written permission to publish XXX under a CC BY license and complete the attached form.”

Please upload the completed Content Permission Form or other proof of granted permissions as an "Other" file with your submission.

In the figure caption of the copyrighted figure, please include the following text: “Reprinted from [ref] under a CC BY license, with permission from [name of publisher], original copyright [original copyright year].”

b. If you are unable to obtain permission from the original copyright holder to publish these figures under the CC BY 4.0 license or if the copyright holder’s requirements are incompatible with the CC BY 4.0 license, please either i) remove the figure or ii) supply a replacement figure that complies with the CC BY 4.0 license. Please check copyright information on all replacement figures and update the figure caption with source information. If applicable, please specify in the figure caption text when a figure is similar but not identical to the original image and is therefore for illustrative purposes only.

The following resources for replacing copyrighted map figures may be helpful:

USGS National Map Viewer (public domain): http://viewer.nationalmap.gov/viewer/

The Gateway to Astronaut Photography of Earth (public domain): http://eol.jsc.nasa.gov/sseop/clickmap/

Maps at the CIA (public domain): https://www.cia.gov/library/publications/the-world-factbook/index.html and https://www.cia.gov/library/publications/cia-maps-publications/index.html

NASA Earth Observatory (public domain): http://earthobservatory.nasa.gov/

Landsat: http://landsat.visibleearth.nasa.gov/

USGS EROS (Earth Resources Observatory and Science (EROS) Center) (public domain): http://eros.usgs.gov/#

Natural Earth (public domain): http://www.naturalearthdata.com/

10. Please upload a new copy of Figures 1, 8 and 9, as the detail is not clear. Please follow the link for more information:  https://journals.plos.org/plosone/s/figures

11. If the reviewer comments include a recommendation to cite specific previously published works, please review and evaluate these publications to determine whether they are relevant and should be cited. There is no requirement to cite these works unless the editor has indicated otherwise.

[Note: HTML markup is below. Please do not edit.]

Reviewers' comments:

Reviewer's Responses to Questions

**Comments to the Author**

1. Is the manuscript technically sound, and do the data support the conclusions?

The manuscript must describe a technically sound piece of scientific research with data that supports the conclusions. Experiments must have been conducted rigorously, with appropriate controls, replication, and sample sizes. The conclusions must be drawn appropriately based on the data presented.

Reviewer #1: Partly

Reviewer #2: Partly

**********

2. Has the statistical analysis been performed appropriately and rigorously?

Reviewer #1: Yes

Reviewer #2: Yes

**********

3. Have the authors made all data underlying the findings in their manuscript fully available?

The PLOS Data policy requires authors to make all data underlying the findings described in their manuscript fully available without restriction, with rare exception (please refer to the Data Availability Statement in the manuscript PDF file). The data should be provided as part of the manuscript or its supporting information, or deposited to a public repository. For example, in addition to summary statistics, the data points behind means, medians and variance measures should be available. If there are restrictions on publicly sharing data—e.g. participant privacy or use of data from a third party—those must be specified.

Reviewer #1: No

Reviewer #2: Yes

**********

4. Is the manuscript presented in an intelligible fashion and written in standard English?

PLOS ONE does not copyedit accepted manuscripts, so the language in submitted articles must be clear, correct, and unambiguous. Any typographical or grammatical errors should be corrected at revision, so please note any specific errors here.

Reviewer #1: Yes

Reviewer #2: Yes

**********

5. Review Comments to the Author

Please use the space provided to explain your answers to the questions above. You may also include additional comments for the author, including concerns about dual publication, research ethics, or publication ethics. (Please upload your review as an attachment if it exceeds 20,000 characters)

**Reviewer #1:** General Comments

This manuscript focuses on the critical issue of high accident rates on mountainous highway curves, and proposes an improved Intelligent Driving Model (IDM) that integrates driving behaviour heterogeneity and curve radius parameters. The authors collected real-world vehicle trajectory data via drones, conducted driving style classification using a DE-optimised K-Means algorithm, and calibrated and validated the model for different driving styles. The research topic aligns with the practical engineering needs of mountainous highway traffic safety, and holds certain theoretical significance and application potential for micro traffic flow simulation and intelligent driving system design for mountainous roads. However, there are several key issues in the manuscript regarding the demonstration of research novelty, mechanistic explanation of the improved model, rigour of conclusions, and writing standardisation, which require major revisions before further consideration for publication.

Major Comments

Comment 1: Further optimise the demonstration of research novelty and clearly define incremental contributions relative to existing literature

While the manuscript claims to "couple driving behaviour characteristics with curve radius into the IDM", existing studies have already developed car-following models that take curve-related factors into account, such as driver visual angle on curves, curve alignment, and complex road geometric conditions. Therefore, the manuscript must clearly and explicitly answer the following core questions to highlight its innovations:

1.What is the new theoretical or methodological increment of this study compared with existing curve-related car-following models?

2.What are the specific advantages of the proposed model over existing car-following models that consider driving style heterogeneity?

3.Is the combination of "driving style classification + curve radius correction" proposed in this study a methodological innovation, scenario innovation, or data application innovation? The manuscript needs to make a clear definition and sufficient argumentation.

Comment 2: Supplement the mechanistic explanation of the improved IDM and the clarification of the physical meaning of parameters

The manuscript states that "a curve radius parameter is introduced to correct the desired speed", but lacks sufficient and rigorous explanation for the core modification logic, which is critical to support the scientific validity of the improved model. The following contents must be supplemented in the revised manuscript:

1.The specific action mechanism of curve radius on the desired speed and acceleration terms of the model, including the complete logical chain of how the curve geometric characteristics are mapped to the driver's car-following decision-making process.

2.The theoretical basis for the functional form of the curve radius correction term. It is necessary to clarify whether this form is derived from vehicle dynamics theory, driving perception mechanism, or empirical assumptions, and provide corresponding theoretical derivation or literature support.

3.Whether there is an interaction effect between the curve radius parameter and driving style parameters, and how this interaction is reflected in the model and verified by empirical data.

Comment 3: Avoid over-extrapolation of conclusions and clarify the applicability boundaries of the model

The empirical data of this study are collected from a single typical mountainous curved section in Yunnan Province, which provides a real case for the research, but also brings obvious limitations to the generalisability of the model. The current model may only be applicable to scenarios under "specific road sections, specific traffic composition, and specific environmental conditions", and its extrapolation performance is still limited. For mountainous roads, there may be significant differences in curve radius and gradient combinations, longitudinal gradient conditions, traffic flow composition, traffic control measures, and marking and signage conditions between different sections. The following revisions are required:

1.Clearly define the applicability boundaries of the proposed model in the Discussion or Conclusion section, and objectively explain the applicable scenarios and constraints of the model.

2.Supplement a detailed description of the sample distribution in the Data Collection and Processing section, including the proportion of vehicle types, traffic flow states (free flow, car-following flow, congested flow), and time period distribution of the collected trajectory data.

3.Add a dedicated subsection of Research Limitations in the Conclusion section, explicitly state the limitations brought by the single-section sample, and propose a clear multi-scenario verification plan for future research.

Minor Comments

Comment 4: Further improve the writing standardisation and terminology accuracy of the manuscript

The manuscript has a basic research framework, but there are still many non-standard issues in writing and expression that need to be revised and improved:

1.Unify the core terminology related to car-following behaviour. The manuscript alternately uses "tailgating behaviour", "following behaviour" and "car-following behaviour", which causes ambiguity. It is recommended to uniformly use the standard academic term "car-following behaviour" in the full text, and only use "tailgating behaviour" when specifically referring to dangerous close following behaviour.

2.Standardise the presentation of figures, tables, variables, units, and symbol annotations. For example, correct the incomplete table titles (such as Table 8), inconsistent figure numbering formats, typographical errors in parameter units in tables, and incomplete explanations of formula symbols, to ensure that all variables and units have clear and consistent physical meaning explanations in the full text.

3.Enrich and standardise the description of research limitations and future research directions in the Conclusion section. The current relevant content is too brief and vague, and needs to be expanded into a structured and specific narrative combined with the actual work of this study.

**Reviewer #2:**  1. Supplement the geometric parameters of curves in the study area, technical parameters of UAV data collection, and verification data of trajectory extraction accuracy; improve on-site real-scene photos of the experiment and complete all experimental elements.

2. Add inter-group significance tests between clustering validity evaluation indicators and driving behavior parameters to enhance the statistical rigor and scientificity of data analysis.

3. Derive the coupling theory between curve radius and the IDM model, and improve the explanation of the physical meaning of parameters; supplement comparative verification with classic car-following models for horizontal curves and enhance the analysis of model generalization ability.

4. In data processing, use km/h for speed and m/s for other variables; units shall be standardized and unified.

5. In the conclusion section, avoid repetition with the abstract and systematically sort out the research context; propose specific directions for future research based on model limitations, and clarify engineering application scenarios such as mountainous traffic simulation and intelligent driving system design.

6. Enrich the discussion section by integrating the latest research findings in driving behavior modeling and road safety evaluation; supplement citations of relevant high-quality literature to strengthen the theoretical support of the study. A real-time conflict risk prediction modeling based on causal inference and graph-model: Applied to multi-configuration expressway diversion zones. Transportation Research Part C: Emerging Technologies, 2025, 178, 105219. https://doi.org/10.1016/j.trc.2025.105219

**********

6. PLOS authors have the option to publish the peer review history of their article (what does this mean?). If published, this will include your full peer review and any attached files.

If you choose “no”, your identity will remain anonymous but your review may still be made public.

**Do you want your identity to be public for this peer review?**  For information about this choice, including consent withdrawal, please see our Privacy Policy.

Reviewer #1: No

Reviewer #2: No

**********

[NOTE: If reviewer comments were submitted as an attachment file, they will be attached to this email and accessible via the submission site. Please log into your account, locate the manuscript record, and check for the action link "View Attachments". If this link does not appear, there are no attachment files.]

To ensure your figures meet our technical requirements, please review our figure guidelines: https://journals.plos.org/plosone/s/figures

You may also use PLOS’s free figure tool, NAAS, to help you prepare publication quality figures: https://journals.plos.org/plosone/s/figures#loc-tools-for-figure-preparation.

NAAS will assess whether your figures meet our technical requirements by comparing each figure against our figure specifications.

# Author comment: Author response to Decision Letter 1

**DOI:** 10.1371/journal.pone.0352855.r002

4 Jun 2026

Dear editors and reviewers�

Thank you for taking your valuable time to review and improve our manuscript entitled "A Study on the Car-Following Model for Mountainous Curves Incorporating Driving behaviour Characteristics". We have revised the manuscript based on your comments and suggestions, and are now submitting the revised version for further consideration by the journal.

In response to the first round of revision comments, we have provided a point-by-point response explaining how we have addressed each editor's or reviewer's comments. Meanwhile, based on the suggestions from the editors and reviewers, we have systematically revised the following sections of the manuscript: Introduction, Literature Review, Dataset and Preparation, Methods, Results, Discussion, and Conclusion. The first response letter has been named "The First Response to Reviewers".

Following the editor's second round of feedback, in addition to addressing the three core comments raised, we have further revised the manuscript to enhance its readability and rigor. The corresponding response document has been named "The Second Response to Reviewers".

Following the reviewers' third round of feedback, we have removed all visible trademarks and copyrighted logos from Figure 4 as requested. The specific revisions have been addressed point by point in the third response letter, which has been named "The Third Response to Reviewers".

In three response documents, blue text represents explanatory content, black text represents the reviewers' comments, and red text represents the revised content.All relevant files have been uploaded to the submission system.

We look forward to receiving your assessment and suggestions.

Sincerely,

Longjiao Zhang.

Yunnan Yunling Highway Science and Technology Co., Ltd. Kunming 650000, Yunnan, China.

Email: zljyyhs@outlook.com

Attachment Submitted filename: The Third Response to Reviewers.docx

# Peer review: Decision Letter 1

**DOI:** 10.1371/journal.pone.0352855.r003

**Authors:** Zhihong (Arry) Yao

16 Jun 2026

A Study on the Car-Following Model for Mountainous Curves Incorporating Driving Behavior Characteristics

PONE-D-26-06596R1

Dear Dr. Zhang,

We’re pleased to inform you that your manuscript has been judged scientifically suitable for publication and will be formally accepted for publication once it meets all outstanding technical requirements.

Within one week, you’ll receive an e-mail detailing the required amendments. When these have been addressed, you’ll receive a formal acceptance letter and your manuscript will be scheduled for publication.

An invoice will be generated when your article is formally accepted. Please note, if your institution has a publishing partnership with PLOS and your article meets the relevant criteria, all or part of your publication costs will be covered. Please make sure your user information is up-to-date by logging into Editorial Manager at Editorial Manager® and clicking the ‘Update My Information' link at the top of the page. For questions related to billing, please contact billing support.

If your institution or institutions have a press office, please notify them about your upcoming paper to help maximize its impact. If they’ll be preparing press materials, please inform our press team as soon as possible -- no later than 48 hours after receiving the formal acceptance. Your manuscript will remain under strict press embargo until 2 pm Eastern Time on the date of publication. For more information, please contact onepress@plos.org.

Kind regards,

Zhihong (Arry) Yao, Ph.D.

Academic Editor

PLOS One

Additional Editor Comments (optional):

Reviewers' comments:

Reviewer's Responses to Questions

**Comments to the Author**

1. If the authors have adequately addressed your comments raised in a previous round of review and you feel that this manuscript is now acceptable for publication, you may indicate that here to bypass the “Comments to the Author” section, enter your conflict of interest statement in the “Confidential to Editor” section, and submit your "Accept" recommendation.

Reviewer #2: All comments have been addressed

**********

2. Is the manuscript technically sound, and do the data support the conclusions?

The manuscript must describe a technically sound piece of scientific research with data that supports the conclusions. Experiments must have been conducted rigorously, with appropriate controls, replication, and sample sizes. The conclusions must be drawn appropriately based on the data presented.

Reviewer #2: Yes

**********

3. Has the statistical analysis been performed appropriately and rigorously?

Reviewer #2: Yes

**********

4. Have the authors made all data underlying the findings in their manuscript fully available?

The PLOS Data policy requires authors to make all data underlying the findings described in their manuscript fully available without restriction, with rare exception (please refer to the Data Availability Statement in the manuscript PDF file). The data should be provided as part of the manuscript or its supporting information, or deposited to a public repository. For example, in addition to summary statistics, the data points behind means, medians and variance measures should be available. If there are restrictions on publicly sharing data—e.g. participant privacy or use of data from a third party—those must be specified.

Reviewer #2: Yes

**********

5. Is the manuscript presented in an intelligible fashion and written in standard English?

PLOS ONE does not copyedit accepted manuscripts, so the language in submitted articles must be clear, correct, and unambiguous. Any typographical or grammatical errors should be corrected at revision, so please note any specific errors here.

Reviewer #2: Yes

**********

6. Review Comments to the Author

Please use the space provided to explain your answers to the questions above. You may also include additional comments for the author, including concerns about dual publication, research ethics, or publication ethics. (Please upload your review as an attachment if it exceeds 20,000 characters)

Reviewer #2: Compared with the original manuscript, the authors have made sufficient revisions and improvements. To further enhance the quality of the paper, the specific comments are proposed as follows:

1. The authors need to enhance the description of the rationality of the clustering results. In Section 4.1.1 "Analysis of Clustering Results", the number of clusters is preset to 3. Although I agree with this practice, a more scientific approach would be to compare the clustering results with different numbers of categories. Therefore, the authors need to explicitly clarify the rationality of presetting the number to 3. It is recommended to refer to: Da Xu, NengChao Lyu, Liu Yang. A Proactive Risk Prediction Framework for Cut-In Maneuvers Incorporating Inherent Driving Styles[J]. IEEE Transactions on Intelligent Transportation Systems, 2025, 26(12), 23084-23099.

2. Information on the proportion of samples after clustering is missing. After conducting the sample clustering, the authors did not introduce the quantity and proportion of the three types of samples. This is very important information, which determines the rationality of the clustering algorithm.

3. The standardization of figures and tables needs to be further enhanced. For example, the text in Figure 1 and Figure 13 is illegible, and Figure 14 lacks horizontal and vertical axis labels.

**********

7. PLOS authors have the option to publish the peer review history of their article (what does this mean?). If published, this will include your full peer review and any attached files.

If you choose “no”, your identity will remain anonymous but your review may still be made public.

**Do you want your identity to be public for this peer review?**  For information about this choice, including consent withdrawal, please see our Privacy Policy.

Reviewer #2: No

**********

# Editor report: Acceptance letter

**DOI:** 10.1371/journal.pone.0352855.r004

**Authors:** Zhihong (Arry) Yao

PONE-D-26-06596R1

PLOS One

Dear Dr. Zhang,

I'm pleased to inform you that your manuscript has been deemed suitable for publication in PLOS One. Congratulations! Your manuscript is now being handed over to our production team.

At this stage, our production department will prepare your paper for publication. This includes ensuring the following:

* All references, tables, and figures are properly cited

* All relevant supporting information is included in the manuscript submission,

* There are no issues that prevent the paper from being properly typeset

You will receive further instructions from the production team, including instructions on how to review your proof when it is ready. Please keep in mind that we are working through a large volume of accepted articles, so please give us a few days to review your paper and let you know the next and final steps.

Lastly, if your institution or institutions have a press office, please let them know about your upcoming paper now to help maximize its impact. If they'll be preparing press materials, please inform our press team within the next 48 hours. Your manuscript will remain under strict press embargo until 2 pm Eastern Time on the date of publication. For more information, please contact onepress@plos.org.

You will receive an invoice from PLOS for your publication fee after your manuscript has reached the completed accept phase. If you receive an email requesting payment before acceptance or for any other service, this may be a phishing scheme. Learn how to identify phishing emails and protect your accounts at https://explore.plos.org/phishing.

If we can help with anything else, please email us at customercare@plos.org.

Thank you for submitting your work to PLOS ONE and supporting open access.

Kind regards,

PLOS ONE Editorial Office Staff

on behalf of

Dr. Zhihong (Arry) Yao

Academic Editor

PLOS One
